# Random forest modelling demonstrates microglial and protein misfolding features to be key phenotypic markers in *C9orf72*‐ALS

**DOI:** 10.1002/path.6008

**Published:** 2022-10-20

**Authors:** Olivia M Rifai, James Longden, Judi O'Shaughnessy, Michael DE Sewell, Judith Pate, Karina McDade, Michael JD Daniels, Sharon Abrahams, Siddharthan Chandran, Barry W McColl, Christopher R Sibley, Jenna M Gregory

**Affiliations:** ^1^ Translational Neuroscience PhD Programme, Centre for Clinical Brain Sciences University of Edinburgh Edinburgh UK; ^2^ Centre for Clinical Brain Sciences University of Edinburgh Edinburgh UK; ^3^ UK Dementia Research Institute University of Edinburgh Edinburgh UK; ^4^ Euan MacDonald Centre for Motor Neurone Disease Research University of Edinburgh Edinburgh UK; ^5^ Centre for Discovery Brain Sciences University of Edinburgh Edinburgh UK; ^6^ Human Cognitive Neuroscience‐Psychology, School of Philosophy, Psychology and Language Sciences University of Edinburgh Edinburgh UK; ^7^ Institute of Quantitative Biology, Biochemistry and Biotechnology, School of Biological Sciences University of Edinburgh Edinburgh UK; ^8^ Simons Initiative for the Developing Brain University of Edinburgh Edinburgh UK; ^9^ Institute of Medical Sciences University of Aberdeen Aberdeen UK

**Keywords:** amyotrophic lateral sclerosis, frontotemporal dementia, C9orf72, neuroinflammation, microglia, post‐mortem tissue, TDP‐43, fused in sarcoma (FUS), machine learning, digital pathology

## Abstract

Clinical heterogeneity observed across patients with amyotrophic lateral sclerosis (ALS) is a known complicating factor in identifying potential therapeutics, even within cohorts with the same mutation, such as *C9orf72* hexanucleotide repeat expansions (HREs). Thus, further understanding of pathways underlying this heterogeneity is essential for appropriate ALS trial stratification and the meaningful assessment of clinical outcomes. It has been shown that both inflammation and protein misfolding can influence ALS pathogenesis, such as the manifestation or severity of motor or cognitive symptoms. However, there has yet to be a systematic and quantitative assessment of immunohistochemical markers to interrogate the potential relevance of these pathways in an unbiased manner. To investigate this, we extensively characterised features of commonly used glial activation and protein misfolding stains in thousands of images of post‐mortem tissue from a heterogeneous cohort of deeply clinically profiled patients with a *C9orf72* HRE. Using a random forest model, we show that microglial staining features are the most accurate classifiers of disease status in our panel and that clinicopathological relationships exist between microglial activation status, TDP‐43 pathology, and language dysfunction. Furthermore, we detected spatially resolved changes in fused in sarcoma (FUS) staining, suggesting that liquid–liquid phase shift of this aggregation‐prone RNA‐binding protein may be important in ALS caused by a *C9orf72* HRE. Interestingly, no one feature alone significantly impacted the predictiveness of the model, indicating that the collective examination of all features, or a combination of several features, is what allows the model to be predictive. Our findings provide further support to the hypothesis of dysfunctional immune regulation and proteostasis in the pathogenesis of C9‐ALS and provide a framework for digital analysis of commonly used neuropathological stains as a tool to enrich our understanding of clinicopathological relationships within and between cohorts. © 2022 The Authors. *The Journal of Pathology* published by John Wiley & Sons Ltd on behalf of The Pathological Society of Great Britain and Ireland.

## Introduction

The most common known monogenetic cause of amyotrophic lateral sclerosis (ALS) is an intronic hexanucleotide repeat expansion (HRE) in *C9orf72* [1, 2, 3]. This expansion is associated with diverse clinical presentations, ranging from ALS‐frontotemporal spectrum disorder (ALS‐FTSD) [4] to parkinsonism and psychosis [5, 6, 7, 8, 9, 10]. Because clinical trials in people with ALS often depend on phenotypic outcome measures such as survival and motor and cognitive functional ratings [11], clinical heterogeneity is a complicating factor in identifying potential therapeutics.

Despite clinical differences, *C9orf72* mutations are pathologically similar, exhibiting a p62+ granule cell layer of the hippocampus and cerebellum [6, 12, 13], dipeptide repeat (DPR) protein deposits [14, 15, 16] and aggregates of the RNA‐binding protein (RBP) TDP‐43 [6, 9, 17, 18] in the central nervous system. C9‐ALS can be differentiated from sporadic ALS through TDP‐43−, p62+ staining in regions of the hippocampus and cerebellum [17]. Notably, p62 is currently the only accurate pathological marker used to identify C9‐ALS in UK clinical practice; although DPR proteins and *C9orf72* RNA foci are pathological markers of C9‐ALS, they are not used in routine neuropathology practice. Indeed, although TDP‐43 is considered a hallmark pathological protein in ALS [19], it is also observed in limbic‐predominant age‐related TDP‐43 encephalopathy (LATE) dementia [20, 21] and healthy ageing [22, 23]. It was recently shown that the wildtype form of RBP fused in sarcoma (FUS) mislocalised in sporadic ALS [24]. FUS dysregulation has yet to be observed in C9‐ALS, though research demonstrating association of *C9orf72* with FUS in stress granules and the negative effects of *C9orf72*‐related DPRs on the regulation of RBPs suggests it is possible [25, 26]. Though informative, none of these features account for all clinical heterogeneity observed in C9‐ALS, so further investigation into markers of C9‐ALS and associated symptoms using clinically annotated tissue is warranted.

It has been suggested that inflammatory profiles may influence the manifestation or severity of motor and cognitive symptoms across the clinical spectrum [27, 28, 29, 30]. A recent study observed differing neuroinflammatory profiles in the cerebrospinal fluid (CSF) between participants with ALS, frontotemporal dementia (FTD) and ALS‐FTSD [31]. However, trials exploring the use of anti‐inflammatory drugs for ALS therapy have thus far been unsuccessful [32], potentially due to non‐specific mechanisms of action when a more nuanced, pathway‐specific manipulation is necessary. Interestingly, though glia and glial activation play a central role in neuroinflammatory processes [33], few studies have profiled glial or glial activation staining markers in C9‐ALS post‐mortem tissue [28, 34, 35]. More extensive characterisation of such markers is needed to better understand glia‐related neuroinflammation in C9‐ALS.

We present here the first extensive digital pathological assessment of intensity, morphological and spatial markers to profile glia‐related inflammation and protein misfolding in deeply clinically profiled C9‐ALS and C9‐ALS‐FTSD post‐mortem tissue. We trained a random forest disease classifier using features derived from our automated digital analysis to evaluate markers of C9‐ALS status. Features were then assessed individually to explore relationships with clinical phenotypes.

## Materials and methods

### Ethics approval and consent to participate

All post‐mortem tissue was collected with ethics approval from East of Scotland Research Ethics Service (16/ES/0084) in line with the Human Tissue (Scotland) Act (2006). Use of post‐mortem tissue for studies was reviewed and approved by the Edinburgh Brain Bank ethics committee and the Academic and Clinical Central Office for Research and Development (ACCORD) medical research ethics committee (AMREC). Clinical data were collected as part of the Scottish Motor Neurone Disease Register (SMNDR) and Care Audit Research and Evaluation for Motor Neurone Disease (CARE‐MND) [36] platform, with ethics approval from Scotland A Research Ethics Committee (10/MRE00/78 and 15/SS/0216). All patients consented to the use of their data during life.

### Case identification and cognitive profiling

Tissue from 10 C9‐ALS‐(FTSD) cases was obtained from the Medical Research Council (MRC) Edinburgh Brain Bank (Table 1). Donors underwent neuropsychological testing during life with the Edinburgh Cognitive and Behavioural ALS Screen (ECAS) [11] and consented to the use of their data. Clinical correlates of disease progression include disease duration and sequential ALS functional rating scale (ALSFRS) data points. Clinical correlates of cognition include ECAS scores. Cases underwent whole genome sequencing, and pathogenic *C9orf72* HREs were confirmed by repeat‐primed polymerase chain reaction (PCR). Cases were graded for amyloid‐beta deposition (Thal) [37], neuritic plaque density (CERAD) [38], neurofibrillary tau tangles (Braak) [39], Lewy body disease (Newcastle) [40] and small vessel disease (Edinburgh Brain Bank ordinal scale). Tissue from 10 age‐ and sex‐matched controls was obtained from the Edinburgh Sudden Death Brain Bank. Controls had no evidence of neurodegenerative pathology upon post‐mortem examination.

**Table 1:** C9‐ALS and control cohort demographics.

Case number	Sex	Age at death (*y*)	Clinical diagnoses	Final CNS diagnoses	Disease duration (*m*)	Region of onset	ECAS	ALSFRS
Case 1	F	63	ALS	ALS *C9orf72* repeat expansion; FTLD‐TDP‐43; Braak NFT stage I	25	Lower limb	Yes (unimpaired)	No
Case 2	M	50	ALS	ALS *C9orf72* repeat expansion	29	Bulbar	Yes (language dysfunction)	Yes (4 data points)
Case 3	F	63	ALS	ALS *C9orf72* repeat expansion	30	Upper limb	No	Yes (3 data points)
Case 4	F	62	ALS	ALS *C9orf72* repeat expansion; Mild non‐amyloid SVD; Thal phase 1	37	Mixed	Yes (unimpaired)	Yes (6 data points)
Case 5	F	65	ALS	ALS *C9orf72* repeat expansion	44	Lower limb	No	Yes (2 data points)
Case 6	M	65	ALS	ALS *C9orf72* repeat expansion	50	Upper limb	No	Yes (1 data point)
Case 7	M	62	ALS	ALS *C9orf72* repeat expansion; Alzheimer's disease: Braak NFT stage IV; Occasional cortical Lewy bodies: Newcastle LBD, neocortical subtype	50	Lower limb	Yes (executive dysfunction)	Yes (2 data points)
Case 8	M	43	ALS	ALS *C9orf72* repeat expansion	57	Bulbar	Yes (language dysfunction)	No
Case 9	M	58	ALS	ALS *C9orf72* repeat expansion; mild non‐amyloid SVD	87	Lower limb	Yes (language dysfunction)	Yes (3 data points)
Case 10	F	63	ALS, FTD, Schizophrenia	ALS FTD‐TDP‐43; Probable *C9orf72* abnormality	119	Upper limb	Yes (FTD – 3 domains)	No
Control 1	F	65	Hypertension, Cardiomyopathy	No significant abnormalities	N/A	N/A	N/A	N/A
Control 2	M	50	None	No significant abnormalities	N/A	N/A	N/A	N/A
Control 3	F	57	None	No significant abnormalities	N/A	N/A	N/A	N/A
Control 4	F	57	None	No significant abnormalities	N/A	N/A	N/A	N/A
Control 5	F	71	Depression, Paranoid Schizophrenia	No significant abnormalities	N/A	N/A	N/A	N/A
Control 6	M	58	Depression	No significant abnormalities	N/A	N/A	N/A	N/A
Control 7	F	59	Hypertension	No significant abnormalities	N/A	N/A	N/A	N/A
Control 8	M	44	None	No significant abnormalities	N/A	N/A	N/A	N/A
Control 9	M	63	None	No significant abnormalities	N/A	N/A	N/A	N/A
Control 10	F	61	Depression, MS, hypothyroidism	No significant abnormalities	N/A	N/A	N/A	N/A

Demographics for C9‐ALS cases from Edinburgh Brain Bank and age‐ and sex‐matched controls from Sudden Death Brain Bank are shown, including sex, age at death, clinical diagnosis/es, final CNS diagnosis, disease duration, region of onset, and cognitive (ECAS) and motor score (ALSFRS) availability. For final CNS diagnoses, neuropathology was graded using Thal phases of amyloid‐beta deposition, the Consortium to Establish a Registry for Alzheimer's Disease (CERAD) assessment, Braak staging of tau neurofibrillary tangle (NFT) deposition and the Newcastle Lewy body disease (LBD) rating scale. MND, motor neuron disease; FTD, frontotemporal dementia; MS, multiple sclerosis; CNS, central nervous system; SVD, small vessel disease; NFT, neurofibrillary tangles; LBD, Lewy body disease; ECAS, Edinburgh Cognitive and Behavioural ALS Screen; ALSFRS, ALS Functional Rating Scale.

### Immunohistochemistry

Brain tissue was taken from standardised Brodmann areas (BA) BA4, BA39, BA44 and BA46 and fixed in 10% formalin for a minimum of 72 h. Tissue was dehydrated in an ascending alcohol series (70–100%), followed by three 4‐h xylene washes. Three successive 5‐h paraffin wax‐embedding stages were performed, followed by cooling and sectioning of formalin‐fixed, paraffin‐embedded (FFPE) tissue on a Leica microtome into 4‐μm‐thick serial sections, collected on Superfrost slides (Thermo Fisher Scientific, Waltham, MA, USA). Sections were dried overnight at 40 °C and dewaxed using successive xylene washes, followed by alcohol hydration and treatment with picric acid to minimise formalin pigment and quench lipofuscin. For pTDP‐43, CD68, Iba1 and FUS, antigen retrieval was performed in citric acid buffer (pH 6) in a pressure cooker for 30 min, with immunostaining using the UK National External Quality Assessment Service (NEQAS)‐approved (https://ukneqas.org.uk) Novolink Polymer detection system (Leica Biosystems, Newcastle, UK) and UK NEQAS‐approved anti‐phospho(409–410)‐TDP‐43 antibody (2B Scientific, Oxfordshire, UK) at a 1:4,000 dilution; anti‐CD68 antibody (Agilent, Cheadle, UK) at a 1:100 dilution; anti‐Iba1 antibody (Abcam, Cambridge, UK) at a 1:3,000 dilution; anti‐FUS antibody (Bio‐Techne, Minneapolis, MN, USA) at a 1:500 dilution. For glial fibrillary acidic protein (GFAP), no antigen retrieval step was necessary, with immunostaining performed as above with a UK NEQAS‐approved anti‐GFAP antibody (Agilent) at a 1:800 dilution. Colour was developed using 3,3’‐diaminobenzidine (DAB) as chromogen, and sections were counterstained using haematoxylin, according to standard operating procedures.

### Imaging and digital pathology analysis

Whole tissue sections were scanned at 40× objective magnification (Brightfield) using a Hamamatsu NanoZoomer XR (Hamamatsu Photonics Ltd, Welwyn Garden City, UK). Regions of interest (ROIs) were selected using NDP.view2 viewing software (Hamamatsu): in both grey and white matter, three vascular‐adjacent and three non‐vascular‐adjacent ROIs (500 × 500 μm) were taken per section per stain. Vascular‐adjacent ROIs were centred on a vessel larger than a small capillary, with larger, cross‐sectional vessels captured where possible. Non‐vascular‐adjacent ROIs were at least ~250 μm from any vessel larger than a small capillary (Figure 2B, supplementary material, Figure S1A). ROIs were analysed using QuPath [41] (see Supplementary materials and methods for details). Whole‐slide manual pTDP‐43 grading was performed as described previously [42] and visualised using GraphPad Prism [43] (version 9.2.0; GraphPad Software Inc, San Diego, CA, USA). ROI‐based manual grading was performed on 60% of CD68, Iba1, GFAP and pTDP‐43 ROIs, as described previously [28, 34, 42] for validation of digital Allred scoring, on 100% of BA39 ROIs for pTDP‐43 quantification validation, and visualised using RStudio (R version 4.1.1) [44] with the ggplot2 package (version 3.3.5) [45].

Image‐, cell‐ and superpixel‐level data from digital analyses were visualised in RStudio (R version 4.1.1) with the ggplot2 (version 3.3.5) and Ggally (version 2.2.1) packages [45]. Data sets with normal distributions (Shapiro–Wilk's test) were subjected to unpaired *t*‐tests for two‐group comparisons and ANOVA with Tukey's multiple comparisons correction for three‐group comparisons. Non‐normal data sets were subjected to Mann–Whitney *U* tests for two‐group comparisons, pairwise Wilcoxon with Holm–Šidák multiple comparisons correction for three‐group comparisons and Spearman's test for correlations. Statistical comparisons were only conducted between groups with *n* ≥ 3. Results were averaged over three ROIs from the same region and presented as ungrouped or grouped by region. For boxplots, data were further averaged when regional stratifications were not included to avoid pseudoreplication. For correlations, unless stated otherwise, all data were included (post‐ROI triplicate averaging) even when data were ungrouped to explore relationships between two stains across all ROIs. Multiple linear regression models were fitted where significant correlations were found, and coefficients were compared by *t*‐tests on individual regression coefficients.

### Machine learning

The automated QuPath analysis generated ~80.2 million superpixel features and ~5.2 million cell segmentation features, 56.4 million of which (66%) were used to train random forest classifiers. For this cohort, we also had more in‐depth clinical information; however, given the clinical heterogeneity within a small sample size, we chose to model a ‘disease’ versus ‘control’ classifier and not stratify further based on other clinical variables. Yet, investigation of individual features did consider clinical phenotypic data. Classification of samples was performed using the random forest implementation in Knime 4.4.1 [46]. Classifiers were trained with 1,000 trees per classifier and information gain as the split criterion and tested on the remaining 33% of the data set. Cross‐tables were generated and *𝜒* ^2^ tests performed to assess the predictiveness of stains and regions. Sensitivity (100 × true positives/total disease) and specificity (100 × true negatives/total control) scores were calculated (supplementary material, Table S1). Three‐fold cross‐validation was performed to assess model validity (supplementary material, Table S2). Finally, leave‐one‐out analyses determined the predictive power of each feature (supplementary material, Tables S3 and S4).

### Reverse transcription quantitative PCR

Reverse transcription quantitative PCR (RT‐qPCR) was conducted by extracting RNA from human FFPE tissue using RNAstorm FFPE RNA extraction kits (Cell Data Sciences, Freemont, CA, USA) on two 10‐μm curls per sample cut from BA4. RNA was eluted in 50 μl nuclease‐free water, after which sample concentrations were measured using a NanoDrop 1000 spectrophotometer (Thermo Fisher Scientific) and concentrated further for 10–15 min at 45 °C using an Eppendorf Concentrator Plus (Eppendorf, Hamburg, Germany), as needed. An aliquot (125 ng) of RNA was reverse‐transcribed using Superscript IV (Life Technologies, Carlsbad, CA, USA) following the manufacturer's instructions, after which 10 ng cDNA was amplified using PowerUp SYBR Green Master Mix (Thermo Fisher Scientific) and forward and reverse primers for *FUS* and the reference 18S ribosomal RNA gene *18Sr* (0.5 μm) (see Supplementary materials and methods for details). RT‐qPCR was performed on eight control and eight C9‐ALS samples in 10‐ng triplicates using a qTOWER^3^ 84 (Analytik Jena GMBH, Jena, Germany) (50 °C for 2 min, 95 °C for 2 min, 40 × 95 °C for 5 s, 60 °C for 30 s). Cycle threshold (Ct) values for *FUS* were normalised (∆Ct) to *18Sr* Ct values, and fold changes (2^−∆∆Ct^) were calculated relative to average ∆Ct of controls. Six controls and seven cases that did not exhibit off‐target amplification and were consistent among triplicates were included in the analysis. Conditions were visually inspected for homogeneity of variance, and an unpaired *t*‐test was performed.

## Results

### Digitally and manually graded staining severity scores correlate significantly

**Figure 1:**
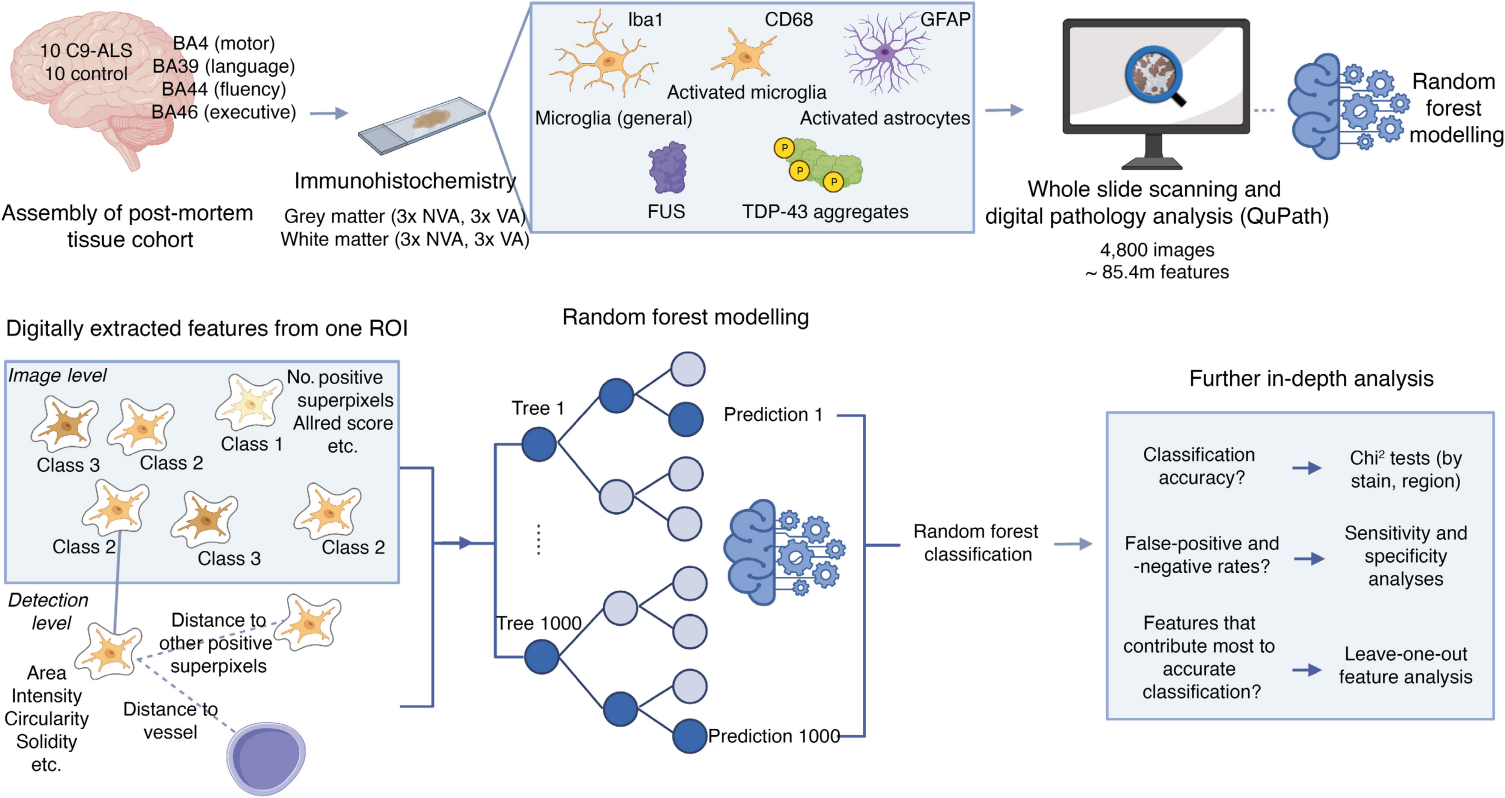
Digital pathology analysis and random forest modelling workflow for characterisation of glial activation and protein misfolding in post‐mortem tissue. Study workflow showing (top) assembly of post‐mortem tissue cohort, staining with a panel of commonly used immunohistochemical stains, whole slide scanning, digital pathology analysis and random forest modelling, and (bottom) example digitally extracted image‐ and detection‐level features, which are used to train and test a random forest classifier, the accuracy of which can be assessed to discern which stains provide the most predictive information about C9‐ALS status. Figure created with BioRender.com. BA, Brodmann area; NVA, non‐vascular‐adjacent; VA, vascular‐adjacent; ROI, region of interest.

**Figure 2:**
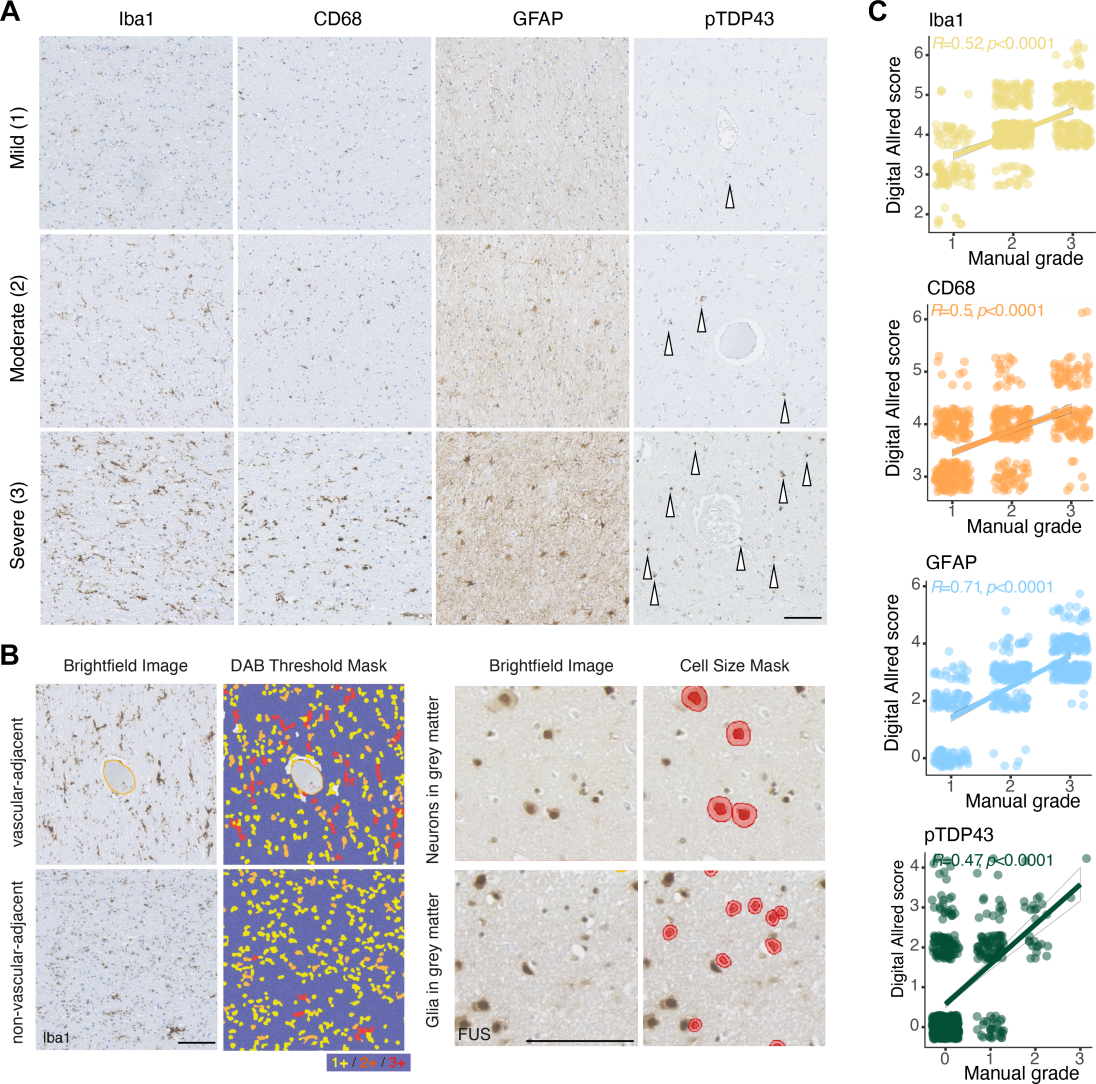
Digital Allred scoring is comparable to manually graded severity scores. (A) Example of stain severities graded on an ordinal scale as mild (1), moderate (2) and severe (3) for Iba1, CD68, GFAP and pTDP‐43. pTDP‐43 aggregates are indicated with white arrows. (B) (left) Example ROIs comprising brightfield images of Iba1‐stained tissue in vascular‐adjacent and non‐vascular‐adjacent regions (Iba1 = brown DAB staining with a haematoxylin counterstain) with digital quantification method using a superpixel segmentation‐analysis mask coding DAB staining intensity from 1+ to 3+ among other intensity‐based, spatial and morphological features and (right) example neuronal and glial cell masks created by nuclear area thresholding with digital quantification method using a cell segmentation‐analysis mask coding DAB staining by cellular compartment among other intensity‐based, spatial and morphological features, used for FUS. (C) Correlations between ROI‐based manual grading and digital Allred score for Iba1, CD68, GFAP and pTDP‐43, demonstrating significant correlations for each stain. All quantified ROIs are included (60% of the data set). Data points are jittered to better illustrate their distribution. Scale bars, 100 μm.

To investigate the pathological differences between C9‐ALS and controls (Figure 1), post‐mortem tissue from a deeply clinically phenotyped cohort (Table 1) was stained with antibodies commonly used in the neuropathological work‐up of ALS cases. Although cases were also examined for other, non‐ALS‐associated neurodegenerative pathology (Table 1), any other neuropathology observed was rare and did not segregate with clinical subtypes. We examined immunoreactivity to microglia‐enriched marker Iba1, macrophage lysosomal activation marker CD68, astrocyte activation marker GFAP, RBP FUS and RBP TDP‐43 (phosphorylated aggregate form) (Figure 1). Stain intensity, morphological and spatial features were obtained in the ALS‐vulnerable motor cortex (BA4) and three FTD‐vulnerable cognitive regions related to the cognitive domains assessed with ECAS [11]; BA39 (language), BA44 (fluency) and BA46 (executive). These were further stratified by vascular adjacency in grey and white matter, resulting in 4,800 images for automated digital analysis, with 240 images per case. Although our aim was to integrate and holistically examine immunohistochemical staining features and their relationship to both each other and to clinical aspects of disease, rather than to compete with manual pathological grading in a diagnostic context, we recognise the value of a manual comparison to demonstrate that a relevant severity score from digital analysis correlates with manual grading to confirm the robustness of digital analysis. We manually graded staining severity [28, 42] in a subset (60%) of ROIs for Iba1, CD68, GFAP and pTDP‐43 (Figure 2A). FUS was excluded from this analysis because staining localisation, and not intensity, is its most biologically relevant aspect. We found that the digital Allred score, which considers the distribution of superpixel stain intensities (Figure 2B), correlated significantly with ROI‐based manual grading (*p* < 0.001) (Figure 2C).

### Microglial and FUS staining features are accurate classifiers of C9‐ALS

**Figure 3:**
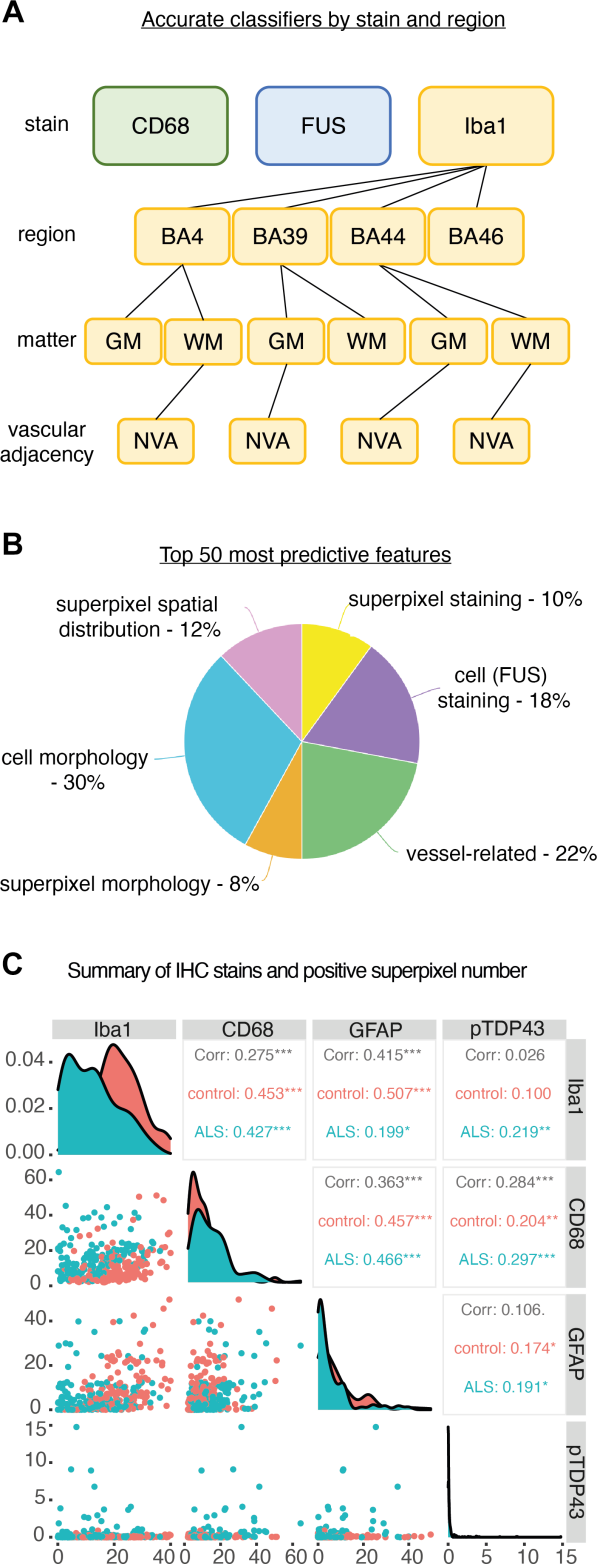
Random forest modelling reveals microglial and FUS immunohistochemical staining features to be accurate classifiers of disease status. (A) Hierarchical organisation of results from random forest analysis demonstrating accuracy of each stain as a disease classifier. Colours represent different stains, lines represent subdivision of brain regions and subregions. For example, Iba1 demonstrates multilevel accuracy at the (i) stain, (ii) brain region, (iii) matter (white matter and grey matter) and (iv) vascular adjacency (vascular‐adjacent and non‐adjacent). (B) Pie chart (created using meta‐chart.com) representing top 50 most predictive features from leave‐one‐out feature analysis, represented in 6 groups related to staining, morphology and spatial distribution. All six feature types are represented in the top 50 most predictive features. (C) Correlation matrix demonstrating relationships between positive superpixel counts for Iba1, CD68, GFAP and pTDP‐43. Correlation coefficients (Corr) and *p*‐values (control, ALS) are shown, demonstrating correlations between stains, with scatterplots (left) and variable distribution (diagonal). BA, Brodmann area; GM, grey matter; WM, white matter; NVA, non‐vascular‐adjacent; VA, vascular‐adjacent. Spearman, *p < 0.05, **p < 0.01, ***p < 0.001, ****p < 0.0001.

The predictive potential of all immunohistochemistry (IHC) features (i.e. intensity, morphological, spatial) was interrogated using a three‐fold cross‐validated random forest model to determine whether C9‐ALS versus control status could be predicted based on staining features, thereby indicating which stains were most suitable for further investigation of individual features (Figure 1; supplementary material, Table S2). Considered together, the five‐stain IHC panel was an accurate disease classifier with 69% sensitivity (*𝜒* ^2^ *p* < 0.0001) and 66% specificity (*𝜒* ^2^ *p* < 0.0001) overall (Figure 3A; supplementary material, Table S1). Staining in each brain region was independently predictive of C9‐ALS status, irrespective of whether the region was clinically involved (supplementary material, Table S1). When broken down by stain, CD68, FUS and Iba1 stains were sensitive (i.e. 67, 64 and 78%, *𝜒* ^2^ *p* < 0.0001, *p* < 0.001, *p* < 0.0001 respectively) and specific (i.e. 60, 69 and 84%, *𝜒* ^2^ *p* < 0.05, *p* < 0.0001, *p* < 0.0001 respectively) when predicting disease status, whereas GFAP staining was sensitive (79%, *𝜒* ^2^ *p* < 0.0001) but not specific (53%, *𝜒* ^2^ *p* = 0.527). Notably, pTDP‐43 staining was specific (64%, *𝜒* ^2^ *p* < 0.001) but not sensitive (51%, *𝜒* ^2^ *p* = 0.8744) to disease status (supplementary material, Table S1). This has been shown previously with respect to cognition [42], and we note that TDP‐43 aggregates can be seen in other non‐ALS conditions [20, 21, 47], in healthy ageing generally [22, 23] and in some controls in this cohort (Table 2 and supplementary material, Figure S1E).

**Table 2:** Manual pTDP‐43 grading of ALS cases.

Case number	Neuronal (N), glial (G)	pTDP‐43 grade				ECAS
		BA4	BA39	BA44	BA46	
Case 1	N	2	1	1	1	Unimpaired (mismatch)
	G	2	1	1	1	
Case 2	N	2	1	1	1	Language
	G	3	1	1	1	
Case 3	N	2	1	1	2	N/A
	G	2	1	1	1	
Case 4	N	1	1	1	1	Unimpaired (mismatch)
	G	2	1	1	2	
Case 5	N	1	0	0	0	N/A
	G	2	1	0	0	
Case 6	N	1	1	1	1	N/A
	G	1	0	0	0	
Case 7	N	2	1	1	1	Executive
	G	0	0	0	0	
Case 8	N	1	1	0	0	Language
	G	2	0	0	0	
Case 9	N	3	1	1	1	Language
	G	3	1	1	1	
Case 10	N	2	3	3	3	All (FTD)
	G	3	3	3	3	
Control 1	N	0	0	0	0	Control
	G	0	0	0	0	
Control 2	N	1	0	0	0	Control
	G	0	0	0	0	
Control 3	N	0	0	0	0	Control
	G	0	0	0	0	
Control 4	N	0	0	0	0	Control
	G	0	0	0	0	
Control 5	N	0	0	0	0	Control
	G	0	0	0	0	
Control 6	N	1	0	0	0	Control
	G	0	0	0	0	
Control 7	N	0	0	0	0	Control
	G	0	0	0	0	
Control 8	N	0	0	0	0	Control
	G	0	0	0	0	
Control 9	N	0	0	0	0	Control
	G	0	0	0	0	
Control 10	N	0	0	0	0	Control
	G	0	0	0	0	

Whole‐slide manual grading of neuronal (N) and glial (G) TDP‐43 burden on an ordinal scale: none (0), mild (1), moderate (2) and severe (3). Cases that are not cognitively impaired in a certain cognitive domain but still exhibit pTDP‐43 staining in the related region are labelled as ‘mismatch’ cases. BA, Brodmann area; ECAS, Edinburgh Cognitive and Behavioural ALS Screen, FTD, frontotemporal dementia.

FUS and CD68 staining were accurate classifiers of disease overall, though this was not maintained within subregions. Iba1 was the most predictive stain, with accurate classification in all four brain regions individually, in most grey and white matter regions within those and even in BA39 non‐vascular‐adjacent grey and white matter, underscoring pronounced C9‐ALS‐related changes to microglia. Leave‐one‐out analysis revealed that no individual feature significantly impacted predictive power, indicating that the collective examination of all or many features is what influences predictiveness (supplementary material, Tables S3 and S4). Features were grouped into six themes, which were all represented in the top 50 most predictive features (Figure 3B).

We next performed correlation analyses to determine the relationship between the number of positive superpixels between stains (Figure 3C). Significant correlations were found between the number of Iba1+ and CD68+ superpixels in C9‐ALS (*R* = 0.427, *p* < 0.001) and controls (*R* = 0.453, *p* < 0.001). Additionally, significant correlations were found between the number of CD68+ and pTDP‐43+ superpixels in C9‐ALS (*R* = 0.297, *p* < 0.001) and controls (*R* = 0.204, *p* < 0.01). The number of GFAP+ superpixels was found to correlate with CD68+ superpixels in C9‐ALS (*R* = 0.466, *p* < 0.001) and controls (*R* = 0.457, *p* < 0.001), demonstrating consistency between glial activation markers. Notably, digital cell recognition analysis revealed this was not due to an increase in cell number (supplementary material, Figure S1B,C). Finally, total GFAP+ superpixels were also found to correlate with pTDP‐43+ superpixels in C9‐ALS (*R* = 0.191, *p* < 0.05) and controls (*R* = 0.174, *p* < 0.05). When visually inspected, the data spread was most distinct between C9‐ALS and controls for Iba1+ versus CD68+ and CD68+ versus pTDP43+, the details of which are further characterised in Figure 5.

### Changes to FUS localisation are observed in C9‐ALS

**Figure 4:**
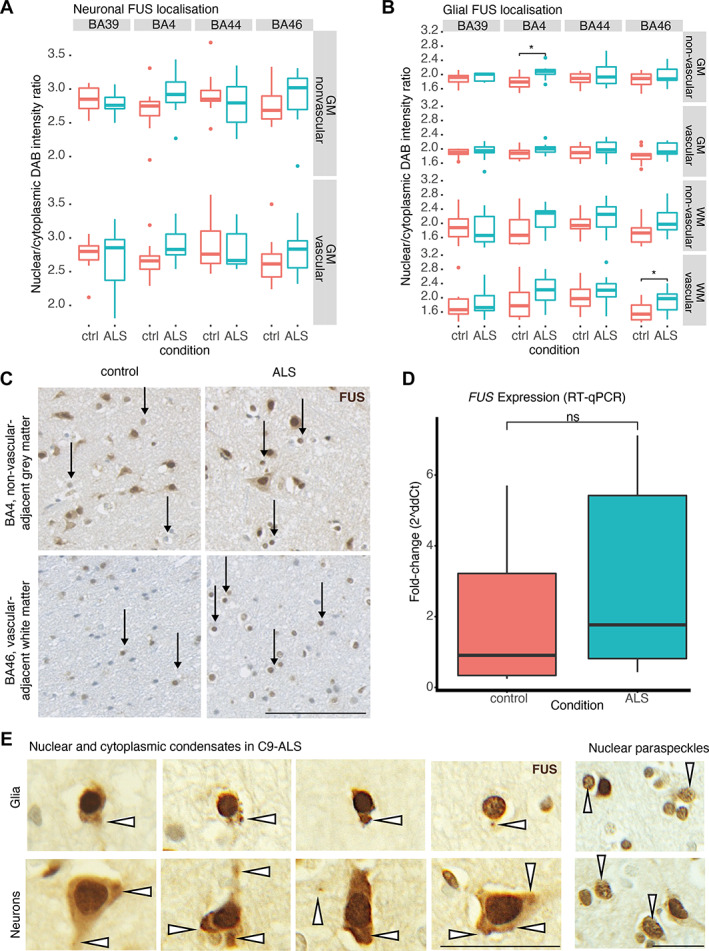
Glia exhibit changes in FUS localisation. Box and whisker plots demonstrating (A) neuronal (unpaired *t*‐test) and (B) glial (Mann–Whitney *U* test) nuclear/cytoplasmic ratio of FUS immunostaining intensity by brain region, demonstrating increased glial nuclear/cytoplasmic FUS ratio in BA4 and BA46 in disease. (C) Representative images of glial FUS staining in regions with significant differences between control and ALS cases from (B). Black arrows indicate glia, demonstrating more prominent glial nuclear FUS staining in ALS compared to controls. Scale bars, 100 μm. (D) RT‐qPCR measurement of *FUS* expression demonstrating no significant difference between control and disease (unpaired *t*‐test). (E) Examples of FUS nuclear and cytoplasmic condensates and nuclear paraspeckles in C9‐ALS cases. Arrows indicate FUS condensates (left) and nuclei with paraspeckles (right). Scale bars, 25 μm. BA, Brodmann area; GM, grey matter; WM, white matter; NVA, non‐vascular‐adjacent; VA, vascular‐adjacent. *p < 0.05, **p < 0.01, ***p < 0.001, ****p < 0.0001.

FUS localisation features appeared to have more predictive power with respect to C9‐ALS status than superpixel count features (Figure 3B). To test whether this stain acted as a proxy measure for neuronal loss, since FUS is a predominantly nuclear protein, we investigated whether there was a reduction in neuronal, glial or superpixel counts for FUS in C9‐ALS. However, no significant reductions were found (supplementary material, Figure S1B–D). Given previous reports of FUS mislocalisation and condensates in sporadic ALS [24], we assessed for digitally apparent differences in localisation and aggregation. There were significant increases in nuclear/cytoplasmic mean intensity ratio in BA4 and BA46 glia, but not neurons (Figure 4A–C). We next interrogated whether this effect was associated with increased *FUS* expression or, rather, nuclear FUS aggregation using RT‐qPCR; there was no significant difference in *FUS* expression between C9‐ALS and controls (Figure 4D). Further support to the notion that this effect could be related to failed degradation and aggregation of FUS is that cytoplasmic condensates in both neurons and glia are readily identifiable in these cases (Figure 4E). Interestingly, when examining serial sections, there are cells and regions in which overlapping FUS and TDP‐43 pathologies can be visualised (supplementary material, Figure S1F,G).

### C9‐ALS post‐mortem tissue exhibits microglia‐related changes

**Figure 5:**
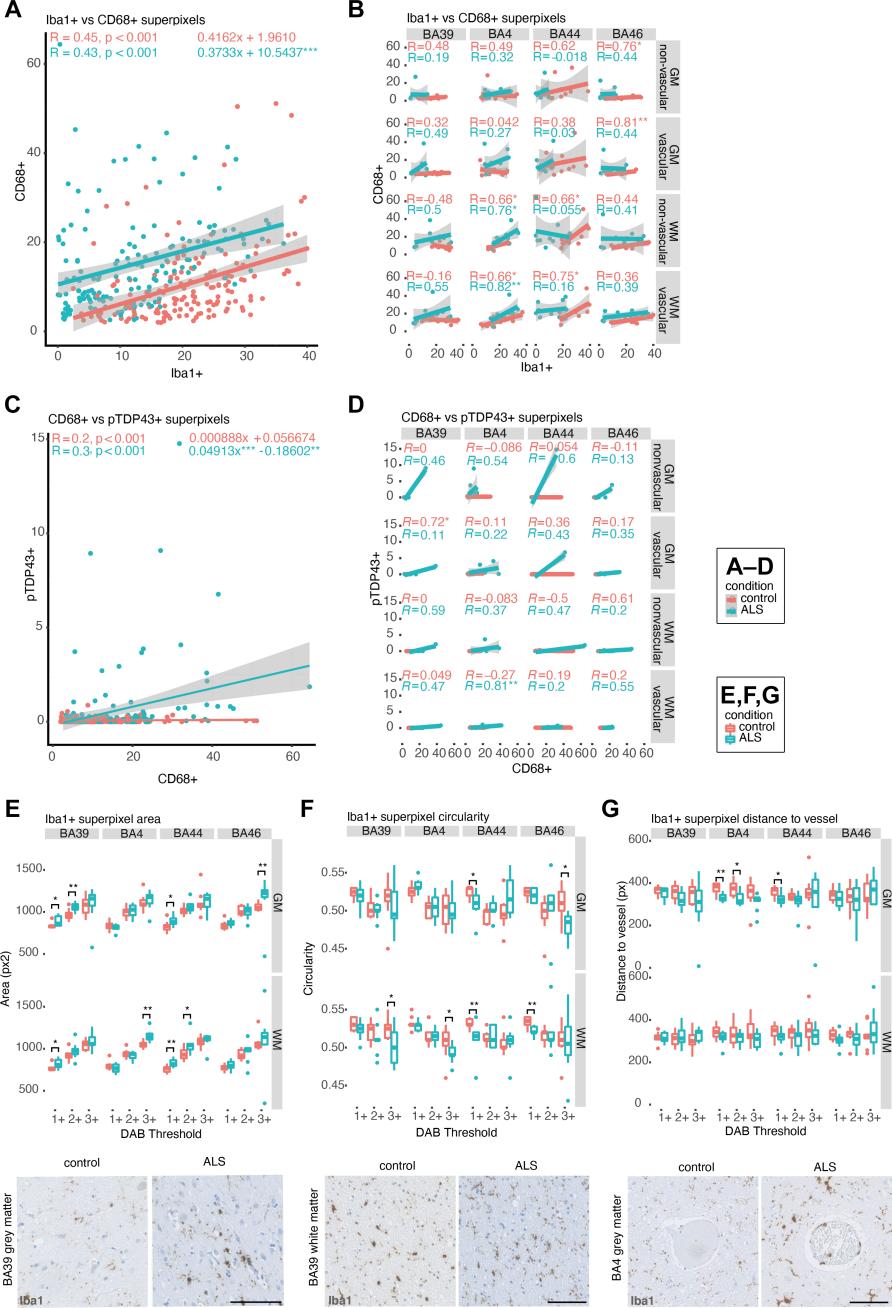
C9‐ALS post‐mortem tissue exhibits microglia‐related changes. (A) Scatterplot of the relationship between CD68+ (activated microglial stain) and Iba1+ (total microglial stain) superpixel number demonstrating an overall upwards shift in disease (taking into account all regions from all cases). (B) Correlation matrix plotting data from (A) by brain region subdivided into GM and WM and vascular‐adjacent and non‐adjacent regions. (C) Scatterplot of the relationship between CD68+ and pTDP‐43+ superpixel number demonstrating an increase in activated microglia staining with increasing pTDP‐43 staining in disease (taking into account all regions from all cases). (D) Correlation matrix plotting data from (C) by brain region subdivided into GM and WM and vascular‐adjacent and non‐adjacent regions. (E–G) Examples of feature analysis for Iba1 staining, demonstrating (E) increased staining intensity, (F) decreased circularity and (G) greater proximity to blood vessels of Iba1+ superpixels in disease, with example DAB images below. Superpixel intensity classes 1–3 represent increasing DAB intensity, with 1 = mild, 2 = moderate, 3 = high. BA, Brodmann area; GM, grey matter; WM, white matter. Spearman (A–D) and Mann–Whitney *U* test (E–G), *p < 0.05, **p < 0.01, ***p < 0.001, ****p < 0.0001. Scale bars, 100 μm.

Further investigation of microglia‐related superpixel correlations with multiple linear regression revealed that *y*‐intercepts (disease: *y* = 10.5437, control: *y* = 1.9610), but not slopes (disease: *a* = 0.3733, control: *a* = 0.4162), of the linear models of Iba1+ versus CD68+ superpixels were significantly different between C9‐ALS and control cases (*p* < 0.001), indicating elevated microglial activation in C9‐ALS (Figure 5A). This pattern was consistent across most regions, and although power was reduced when the data were stratified, significant correlations in disease persisted in BA4 white matter (Figure 5B). For the relationship between CD68+ and pTDP‐43+ superpixel number, multiple linear regression revealed that both slopes (disease: *a* = 0.04913, control: *a* = 0.000888; *p* < 0.001) and *y*‐intercepts (disease: *y* = −0.18602, control: *y* = 0.056674; *p* < 0.01) of the linear models were significantly different between C9‐ALS and controls (Figure 5C), suggesting a relationship between microglial activation and TDP‐43 aggregation in disease. This relationship is preserved particularly across grey matter, as well as in BA4 and BA39 white matter (Figure 5D). However, the only significant correlation in C9‐ALS was found in BA4 vascular‐adjacent white matter.

Analysis of specific Iba1 features revealed significant increases in C9‐ALS versus control Iba1+ superpixel area in BA4, BA39 and BA44 white matter regions and in extra‐motor grey matter (Figure 5E). There were also significant decreases in the circularity of class 1+ and class 3+ superpixels in C9‐ALS, predominantly in white matter (Figure 5F). It must be noted that these findings revealed changes to the area and circularity of superpixels containing Iba1 stain, thereby indicating disease‐related morphological changes that are not necessarily physiological or suggestive of activation status. Finally, significant decreases in the distance of class 1+ and class 2+ superpixels from the central vessel were observed in C9‐ALS, predominantly in BA4 grey matter (Figure 5G), which may reflect migration to vessels as a response to systemic inflammation or compromised blood–brain barrier integrity [48]. It is also possible that the combined result of morphological changes and increased vessel‐adjacent Iba1+ staining is indicative of an increased presence of non‐microglial, Iba1+ monocyte‐derived cells that have migrated from vessels in disease.

### Clinicopathological relationships exist between microglial activation status and cognitive dysfunction

**Figure 6:**
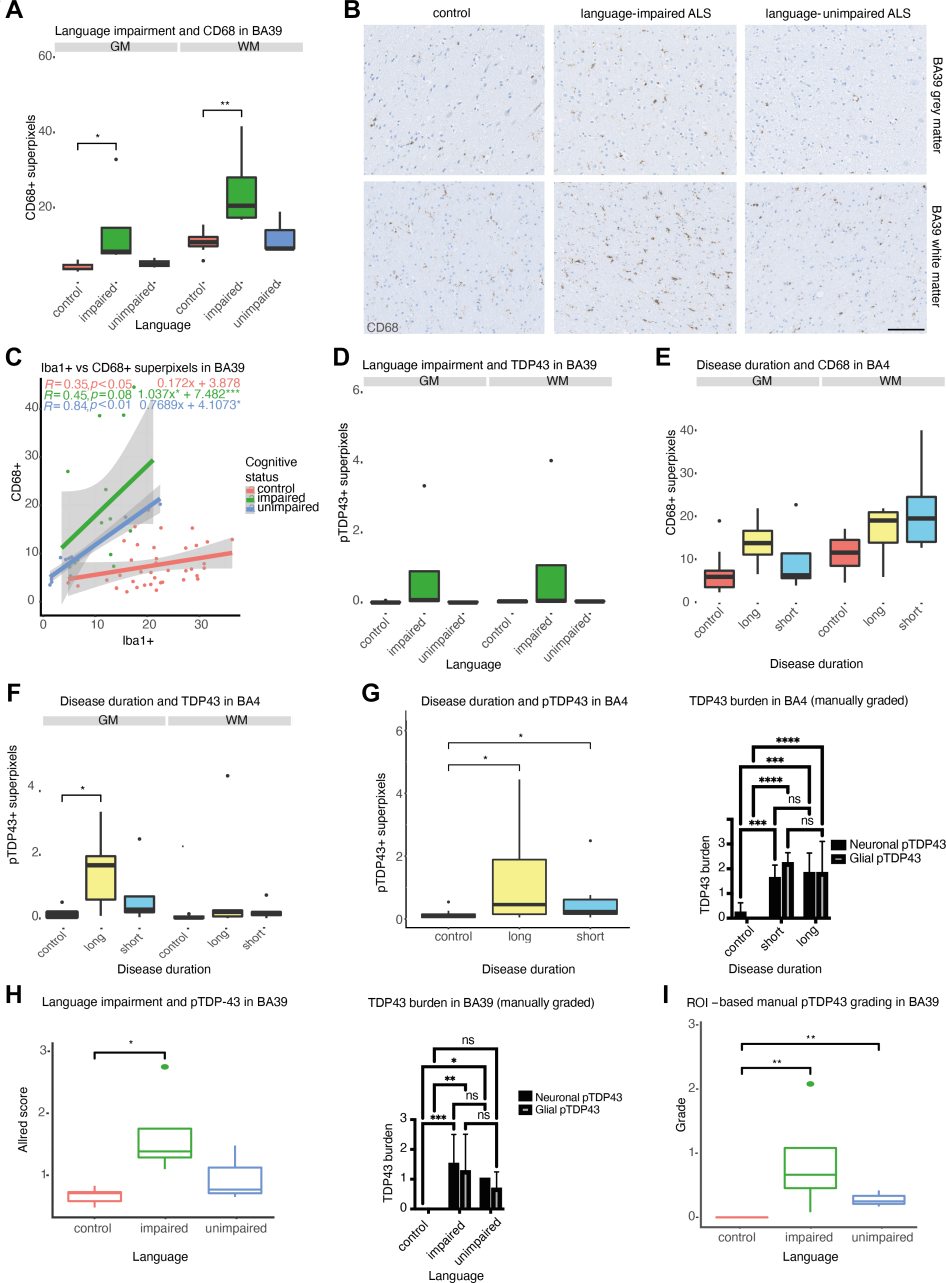
Clinicopathological associations exist between microglial activation status and cognitive dysfunction. (A) Box and whisker plot showing CD68 immunoreactivity in cases grouped by language impairment, demonstrating increased CD68+ superpixel count in language‐impaired cases in BA39. (B) Example images (GM above and WM below) with CD68 immunostaining (brown/DAB) and haematoxylin counterstain showing increased immunostaining in language‐impaired cases in BA39. (C) Scatterplot demonstrating differing relationships between CD68+ and pTDP‐43+ superpixel count in BA39 between control and ALS cases stratified by cognitive status. (D) Box and whisker plot showing pTDP‐43 immunoreactivity in cases grouped by language impairment, demonstrating increased (non‐significant) pTDP‐43+ superpixel count in language‐impaired cases in BA39. (E) Box and whisker plot showing CD68+ superpixel count in BA4 cases grouped by disease severity (i.e. disease duration from symptom onset), with no significant differences found. (F) Box and whisker plot showing pTDP‐43 immunoreactivity in BA4 of cases grouped by disease severity, demonstrating increased pTDP‐43+ superpixel count in GM of long survivors. (G) Box and whisker plots showing comparison between (left) digital analysis (Wilcoxon, Holm–Šidák test) of pTDP‐43+ superpixels and (right) manual whole‐slide pTDP‐43 grading (ANOVA, Tukey's test) by a ‘blinded’ pathologist in BA4 in cases stratified by disease duration. (H) Box and whisker plots showing comparison between (left) digital analysis (Wilcoxon, Holm‐Sidak test) of pTDP‐43+ Allred score and (right) manual whole‐slide pTDP‐43 grading (ANOVA, Tukey's test) by a ‘blinded’ pathologist in BA39 in cases stratified by language impairment status. (I) Box and whisker plot of manual ROI‐based pTDP‐43 grading in BA39 (Wilcoxon, Holm–Šidák test). Data in box and whisker plots are averaged across quantified regions not included in presented stratification, such that each category contains *n* = number of cases, to avoid pseudoreplication. BA, Brodmann area; GM, grey matter; WM, white matter. Spearman, Wilcoxon with Holm–Šidák, *p < 0.05, **p < 0.01, ***p < 0.001, ****p < 0.0001. Scale bars, 100 μm.

IHC findings were next analysed for relationships with clinical features by comparing cognitively unimpaired C9‐ALS cases to C9‐ALS‐FTSD cases. Language‐impaired cases (*n* = 4) were found to have significantly more CD68+ superpixels in BA39 grey matter compared to controls (*n* = 10) in both grey (*p* < 0.05) and white (*p* < 0.01) matter, whereas this was not the case for language‐unimpaired cases (*n* = 3) (Figure 6A–C). Both C9‐ALS subgroups (language‐impaired and language‐unimpaired) had more CD68+ superpixels per Iba1+ superpixel in BA39, suggestive of elevated microglial activation in C9‐ALS, with significant differences in *y*‐intercepts of linear models (language‐impaired: *y* = 7.482, *p* < 0.001; language‐unimpaired: *y* = 4.1073, *p* < 0.05) compared to controls (*y* = 3.878) (Figure 6C). The slope of the linear model for language‐impaired cases was significantly increased compared to controls (impaired: *a* = 1.037, control: *a* = 0.172, *p* < 0.05), whereas this was not the case for language‐unimpaired cases (*a* = 0.7689), potentially suggesting a more reactive microglial phenotype in impaired cases (Figure 6C). Further, there were no significant differences in pTDP43+ superpixel count in BA39 grey or white matter detected between language‐impaired, language‐unimpaired and control conditions (Figure 6D). No significant differences in CD68+ superpixel number were found between long survivors (i.e. >48 months [49]), short survivors and controls (Figure 6E). Total pTDP‐43+ superpixel count was significantly increased in long survivors in BA4 grey matter (*p* < 0.05) compared to controls, which may indicate a build‐up of aggregates over time (Figure 6F). Finally, no significant differences in total GFAP+ superpixels were detected between the aforementioned clinical groupings and controls (supplementary material, Figure S2C,D).

### The most accurate pTDP‐43 grading features depend on level of burden

Considering that TDP‐43 aggregation is a pathological hallmark of ALS [19], it could appear counterintuitive that pTDP‐43 staining did not accurately distinguish C9‐ALS from controls in our model. As such, we next sought to further evaluate digital (i.e. included in the model) and manual methods of pTDP‐43 quantification in BA4 (Figure 6G) and BA39 (Figure 6H). TDP‐43 aggregate burden was manually graded [42] across whole slides for comparison with digital positive superpixel count (Table 2). In BA4, which contained the highest TDP‐43 burden across cases, significant differences in neuronal and glial burden between C9‐ALS and controls were picked up by both automated and manual methods, though neither method showed a significant difference between long and short survivors (Figure 6G). Second, due to the nature of automated image analysis and ROI selection, we postulated that a more appropriate digital measurement than positive superpixel count for the milder and sparser TDP‐43 pathology seen in BA39 might be the Allred score, which considers both superpixel intensity and number. Indeed, there was a significant difference in digital Allred score for BA39 pTDP‐43 staining between language‐impaired cases and controls (Figure 6H). In addition, manual grading also detected significant differences in the BA39 TDP‐43 burden between language‐unimpaired cases and controls (Figure 6H). These findings suggest that the most appropriate quantification method may depend on the sparsity of pathology. To interrogate whether ROI‐based sampling influenced quantification differences, pTDP‐43 staining in ROIs from digital analysis was manually graded; both whole‐slide and ROI‐based manual grading detected similar differences between conditions (Figure 6I). All methods were comparable in their ability to discern differences in pTDP‐43 staining, or lack thereof, between C9‐ALS clinical phenotypes. Importantly, an advantage of the digital method is the acquisition of intensity, morphological and spatial features quantified on a continuous scale, providing a level of characterisation that cannot be practically achieved with manual grading.

## Discussion

Here we have outlined a standard for extensive quantitative assessment of neuropathological elements in ALS, building upon previous methods analysing FUS and splicing factor proline‐ and glutamine‐rich (SFPQ) in sporadic ALS [50], and Alzheimer's disease pathology [51, 52]. Importantly, this study allowed for post‐mortem tissue staining to be directly compared with motor and cognitive data. Our model revealed that Iba1, CD68 and FUS staining were more predictive of disease status than pTDP‐43 or GFAP staining. Further investigation revealed a clinicopathological link between microglial activation and both TDP‐43 pathology and language impairment.

Microglial dysregulation in C9‐ALS may be a consequence of haploinsufficiency resulting from *C9orf72* HRE. Microglia have previously been shown to strongly express C9orf72 protein and may therefore be particularly susceptible to C9orf72 depletion secondary to haploinsufficiency [53, 54, 55]. Indeed, *C9orf72* ^−/−^ peripheral myeloid cells have hyperactive type I interferon responses [55], suggesting that loss of normal C9orf72 function can lead to a proinflammatory state. Mouse *C9orf72* ^−/−^ microglia have also been shown to have increased expression of interferon as well as other activation response genes [56]. In humans, both qualitative [35] and semi‐quantitative [34] pathological assessment, as well as the digital analysis, have revealed signs of increased microglial activation related to *C9orf72* HRE. Additionally, CSF from C9‐ALS but not sporadic ALS patients was shown to have significantly higher levels of pro‐inflammatory cytokines and chemokines compared to controls [57]. Our study in particular builds upon these findings by quantifying intensity, morphological and spatial features of microglia, demonstrating a correlation between CD68+ and pTDP‐43+ staining and suggesting a role for extra‐motor microglial activation in cognitive impairment. It may be that alternate TDP‐43 aggregate conformations lead to differential activation of microglia, similar to what has been observed in response to amyloid plaques [58, 59]. Recent studies have demonstrated the immunogenic capacity of TDP‐43 and its ability to activate microglia specifically [60, 61, 62], although further research is needed to characterise aggregate subtypes or ‘strains’ and their effects on microglia.

Finally, our model identified FUS‐related dysregulation in C9‐ALS. FUS is known to undergo liquid–liquid phase separation (LLPS) and associate with membraneless organelles, some of which, such as paraspeckles and nuclear gems, are localised to the nucleus [63]. FUS is also known to autoregulate and increase its own expression as a result of impaired nuclear import [64]. We did not find significant differences in *FUS* expression between controls and C9‐ALS, in support of the former mechanism. It has been shown that functional C9orf72 protein is required for the removal of aggregation‐prone FUS protein [25] and that *C9orf72*‐related DPRs induce aggregation of TDP‐43, which, similar to FUS, has an intrinsically disordered C‐terminal region [65]. Thus, *C9orf72* haploinsufficiency or the interaction of FUS with DPRs may result in impaired FUS degradation; our observations of cytoplasmic condensate and paraspeckle formation in the absence of increased *FUS* expression further support this hypothesis. Finally, the observed co‐occurrence of FUS and TDP‐43 pathology in cells and regions across serial sections suggests joint dysregulation of multiple RBPs; future studies may investigate RBP targeting therapies [66] to discern whether targeting of one RBP is sufficient to prevent a dysregulation cascade or whether the targeting of multiple RBPs is required.

Some limitations should be noted regarding digital analysis. For example, preset staining thresholds in automated digital analysis circumvent the possibility of bias that arises with manual grading, though they may not be as accommodating of staining variation. However, we have limited potential issues with respect to this, as all tissue was banked in the same manner and fixed for the same length of time, and all antibodies and reagents were UK NEQAS‐approved for use in a clinical setting, indicating high consistency between uses. For FUS staining, the QuPath cell segmentation algorithm is less robust than the superpixel segmentation algorithm in brightfield images; to avoid frequent detection of non‐existent cells, haematoxylin thresholds for nuclei identification were set relatively high, resulting in some cells being excluded from the analysis (Figure 2B). Additionally, this study utilised manually selected ROIs based on predetermined criteria, rather than entire tissue scans, to equally represent different regions of interest as part of an exploratory analysis. Thus, it may be worth assessing larger regions in the future, though this would require more sophisticated cortical annotation algorithms than are currently available. Finally, our C9‐ALS cohort was assembled based on the extent of clinical information (e.g. ECAS scores) available to allow for phenotypic comparisons, resulting in a small sample size, though new deeply clinically phenotyped cases may be meta‐analysed with data from the current study as they become available to increase the power of the analyses. Our cohort also did not include multiple fluency‐ or executive‐impaired cases, so we were not able to conduct statistical comparisons regarding impairment in these domains (supplementary material, Figure S2A,B). Although our model provides information about important pathways in C9‐ALS, it is currently unclear whether predictive features are specific to C9‐ALS, or common to all ALS or neurodegeneration in general. Features from other disease cohorts would need to be added in the future to discern this.

Taken together, our high‐throughput digital pathology and random forest modelling approach enabled detailed mapping and holistic analysis of staining features to characterise neuroinflammation and protein dynamics across a clinically heterogeneous C9‐ALS cohort. The identification of these predictors holds promise for peripheral biomarkers and positron emission tomography (PET) ligand creation in the future and contributes to the essential understanding of disease‐related specific pathways for the development of targeted therapeutics. For example, this non‐biased study identified a relationship between neuroinflammation and cognitive phenotype, raising the issue of appropriate trial stratification and endpoints. Clinical trials testing anti‐inflammatory therapies to date have either tested ALS non‐specifically or used ALSFRS and survival as primary endpoints [67]; our findings suggest that stratification on a molecular level based on inflammatory signatures, as well as the use of cognitive in addition to motor endpoints, may be necessary to more meaningfully measure clinical outcomes. Our study also demonstrates the strengths of utilising a standardised digital pathological assessment, from which results can be shared and meta‐analysed to provide a comprehensive and highly powered model revealing pathological predictors of disease and clinical phenotypes. The generation of large data sets using this method allows for the investigation of numerous neuropathological questions, many of which are outside the scope of this study. For this reason, all the scripts and data featured in this study are included in the supplementary information.

## Author contributions statement

OMR, JL, MDES, MJDD, SA and JMG conceived the study. OMR, JO, MDES, JP, KM and JMG acquired the data. OMR, JL, MDES, MJDD, CRS and JMG analysed the data. OMR, JL, JO, MDES, MJDD, JP, KM, SA, SC, BWM, CRS and JMG interpreted the data. OMR, JL, JO, MDES and JMG wrote the original manuscript. OMR, JL, JO, MDES, MJDD, JP, KM, SA, SC, BWM, CRS and JMG revised the manuscript. All authors had final approval of the submitted and published versions.

## Supporting information


Supplementary materials and methods
**Figure S1.** Supplementary digital pathology analysis information
**Figure S2.** Additional glial activation staining
**Table S1.** Random forest classification of stain panel with sensitivity and specificity scores
**Table S2.** Three‐fold cross‐validation of random forest model
**Table S3.** Random forest leave‐one‐out feature analysis
**Table S4.** Random forest features included in model

## Data Availability

The data sets supporting the conclusions of this article are included in this published article and its supplementary material or available in the figshare repository (raw images, digital pathology features, random forest data), https://figshare.com/projects/Random_forest_modelling_and_neuropathological_features_of_a_large_cohort_of_C9orf72‐ALS_all_raw_data_/128222 [68]. Link to raw images: https://figshare.com/articles/figure/C9‐ALS_and_control_images/17145896. Link to digital pathology features: https://doi.org/10.6084/m9.figshare.17145902.v1. Link to random forest results: https://figshare.com/articles/dataset/Random_forest_results/17145905. The SD numbers of cases from the Edinburgh Brain Bank included in the study are available upon request.
