# Supplementary material for: Random forest modelling demonstrates microglial and protein misfolding features to be key phenotypic markers in C9orf72‐ALS
Source: J Pathol. 2022 Oct 20;258(4):366–81. doi: 10.1002/path.6008 (PMC9827842; doi:10.1002/path.6008)
Supplement: Supplementary file 1 — Supplementary materials and methods Figure S1. Supplementary digital pathology analysis information Figure S2. Additional glial activation staining Table S1. Random forest classification of stain panel with sensitivity and specificity scores Table S2. Three‐fold cross‐validation of random forest model Table S3. Random forest leave‐one‐out feature analysis Table S4. Random forest features included in model [file PATH-258-366-s001.docx]

**Random forest modelling demonstrates microglial and protein misfolding features to be key phenotypic markers in *C9orf72*-ALS**

OM Rifai *et al. J Pathol* DOI: [10.1002/path.6008](https://doi.org/10.1002/path.6008)

**Supplementary materials and methods**

**Supplementary Figures S1,S2**

**Supplementary Tables S1–S4**

Reference numbers refer to the main text list

**Supplementary materials and methods**

FUS *RT-qPCR*

Forward and reverse primers to *FUS* and reference 18S ribosomal RNA gene *18Sr* (0.5 µM) were selected to control for age- and degradation-related artefacts. *FUS* primers were designed to target non-contiguous central portions of the sequence so as to detect product that may be partially degraded due to post-mortem autolysis or the nature of FFPE tissue storage.

*18Sr* Forward: 5’-GGCCCTGTAATTGGAATGAGTC-3’

*18Sr* Reverse: 5’-CCAAGATCCAACTACGAGCTT-3’

*FUS* Forward: 5’-ACTTCCTTTCTTTTGCTCTCAC-3’

*FUS* Reverse: 5’-ATTAAGCCCCACTAAGCCC-3’

Primers that failed to amplify *FUS* in FFPE tissue samples, targeting two non-contiguous central regions (R1, R2) listed below. Note that R2P3 showed some amplification but was not as consistent between triplicates as the primer pair used in the study (R2P2).

Region 1

GTTCACACGTGGTGGCATGAAAAGAGTGGCTAAAGTGGTATCAAGACTGCCTGGATGTTCTTTGAAACTA

TTATAAAAAGGAAACTGAAAAAAATGGGGATAGAGAAGGAAGGGAGTTAGGTGTGTCCTTAGTTAGCAGT

GAGAAGTATTTGTTACGAAGTATTTCTCAGAAATACCTGGCTTGTGGGTTCCACCCCCAGTGATTTAGGT

CTGAGAGGACCCTGAAAATCTACCTTTCTAACAAGTCCCCAGTGATGCTGATGCGTCTGGACCACACTCA

GATGGTTTACAGCAGTGGTTCTTTCAAAATGTGGATCATGTCCAAGTTGGTGAACAAAACTAGTGAAAGA

CCTGACCACATGAAGTAAAGCATTGAACTCCTGTTTAGGTTGTATTGATGTTTGTGTACTAGATTTGAAT

GTAAAATGGGTTTCTTATTTAATTCGGGGACCTTCAACTGAAAGTTGATAAATACTGATGGACTTTTCTG

TGTGGTCTTGTTGGGCATTTCACTCCTGAGCCCTGGTTTCCATACTGTATACTGGGTGTTAACATGTTTC

AAAGGATAATTGTCAAACTGAATCTGAAATTTATCAGCATGGCTGGCATATAGGGACTCAAAAGGGATGT

GGATTTCTTTTTAGTTGTCTTCCATAAACCAAATGATACCAGTTGCTTGATGGATACTAGGTGCTTTAGG

TTTTTTCCTGTGTTTTTTATTTTACCTTTTCACATTTGCATTTTCTCTGTTCAACAAGCAGAACAGGATA

ATTCAGACAACAACACCATCTTTGTGCAAGGCCTGGGTGAGAATGTTACAATTGAGTCTGTGGCTGATTA

CTTCAAGCAGATTGGTATTATTAAGGTACTTGTGGAGAGGAGTGGGAGCTTTCTGTCAGTGTTGTAGGCT

TGTGGATTTCACACATTAGTAAAAGCAAGTCTTTAATGGTTGCCAGCAGTAAAAACAAGTCTTAGTGGTT

GTTGCCAGCTTAATTTGTTGAGGAAAGAGCCTTAGTTACTGTTTTCTAAAAGAGAAGTTCTATCTTAACA

CAAAAAGTATAACTTATCAGAGTACCCTAAACTCTTGAGATTTGTACTCTATAGTAACTTTTAGTTTTAT

CTTTCAATATTGGAGTGAGAGACAGTTTTCTTTAATGGAGGTTTACATGTGAGGTAGGAAGAAGTAACTG

GGAAGAGGGGAGCTGAAGTTTGGGAATTATAAACCTCATGTTCTAGAGGAAGAAGATGGAAAGGGAGTAC

TGTAGCCTTTAAAATTGATGTTACCTCATTTTGCTTTCTTCAGACAAACAAGAAAACGGGACAGCCCATG

ATTAATTTGTACACAGACAGGGAAACTGGCAAGCTGAAGGGAGAGGCAACGGTCTCTTTTGATGACCCAC

CTTCAGCTAAAGCAGCTATTGACTGGTTTGATGGTATGTATGAGAAGGCTGGCAGAGGTGGGGCTGGGGA

TATAGGGCAGCAAGCCTTAGGAAACAAGCCATAGTTTGAGGGTTCTTTTGAGTCTTCCAACACTTACTTT

Region 2

GTTGGCCAGGCTGGTCTCGAACTCCTGACCTCGTGATCCACACGCCTCGGCCTCCCAAAGTGCTGGTATT

ACAGGCGTGAGCCACCGCACCCGGTCAGCTTTATTATTAAATGTTAACTTCATCTGCTTTGTACACGAAT

GCATACCCAGTGCCCAGAACAGTGCCTGGAACATACTAGGTGCTAAATAAATATTTGTGACTTAATGCAT

GAATAAGGGTGGACTTCCTTTCTTTTGCTCTCACTGGAGAGTTGAACTCTCCTTCCAAAGGCGGTGGGGT

GGATATTGGCATATTCAGGGCCTTTAGGGCTGAAGTCAAGGGCTTAGTGGGGCTTAATTTGTGGGCGGGC

CCAGGGCATGGCCCTCATTGTTTCTCTAGAAAGACGTGTCCAACCCTCAAAGGACCTTCTGAAATCCCGC

TGAAAGGTTAAGTTGGGAAGGAAAACCTGCATGCCTATGTATCAGGAATTAACGTCTTTTGTCTTGTTTT

ATTCAGTAGTTTCAAATTGCCTTCTCCAGGCAAGGCTGATGAAAGTGCAAGTTGCAAGTTAATTTGAATG

TTTCTTTTTGCTTTTGCTCTCACAGGAAGTGAAAAGGCGACCAAACACTCTAGCATTCATGCCACCAAAA

AGAGGAGTGTTTTGCAGTTACAAGACCTGGATTCGAATCACGACTCCTCTTAGCTGCCCTGTAATCAGGC

ACAATTACTTGGGTCTCTGAGTCTCACTTTCCTTATCTAGAAAACGGAGGTATCTTTACTTCCTTCGTAA

GACTGATGACAAGGAAATTATCTGTGCATTTTGAAACCACTTAAGCCTTGTACACGTTTTATTTCTGGGA

TCGCCCTGGTAGGGCTTCAGAAAAATAAAAAGGAGGTCCCTGAGAAAAGGCTGGGTACCGTACATCTGAG

GTCAACCCTCTCTGGTCCCAAGGATGGCCTGGGCTGTTCCGCCCCGTGGCTCCCCAGGGGCAAAGCCATG

AGGATCCGGGTGAGAGCCCAGTGCTGGACGAGCCCGGGGCCCAGGGGTCCCGGCCGAAATCCCTGCTGTC

TTTCAGGTCAAACGTCATAATCCCCGAACCCCAGAAAGGCCGAAAGGCAAGGCAACCCTGAAAGACGACG

AAGTCAACCTCAGGGCGCAGGAGAGGGAGGGCCAGTGTGCTGCCGACGAGGGAGGCTGGAGCCGCGGGGA

CGAGGCGCCCCATACAGCGGCAAGAGGGTGGAGGGCAGGAGCTCGCCATCCTGGGTGAAAGCGGGGCCCA

GCGAAGGGGCCCGGCCACAGGAATCTCGGTTCCACCCCGCTACTCCCGGCTGTGACTCCAGTTTCGTCCC

CAGCCGCCGGGACCGCCCCCTCGCCCCGCCCCCAGCGGGCACTCAGGCCGTACCACTGTGCCTTCATGGG

R1P1 Forward: 5’-TGGGGATAGAGAAGGAAGGGAG-3’

R1P1 Reverse: 5’-TCCAGACGCATCAGCATCAC-3’

R1P2 Forward: 5’-AATGGGGATAGAGAAGGAAGG-3’

R1P2 Reverse: 5’-AAGAACCACTGCTGTAAACC-3’

R1P3 Forward: 5’-TGGGGATAGAGAAGGAAGGG-3’

R1P3 Reverse: 5’-AAGAACCACTGCTGTAAACC-3’

R2P1 Forward: 5’-ACTTCCTTTCTTTTGCTCTCAC-3’

R2P1 Reverse: 5’-TTCCTTCCCAACTTAACCTTTC-3’

R2P3 Forward: 5’-ACTTCCTTTCTTTTGCTCTCAC-3’

R2P3 Reverse: 5’-TAAGCCCTTGACTTCAGCC-3’

*Further details for QuPath analysis*

Images were segmented into superpixels with stain-specific segmentation parameters selected to best identify the staining of interest (downsample = 1, gaussian sigma = 8.0 for Iba1 and GFAP, gaussian sigma = 5.0 for CD68, FUS and TDP-43). For superpixel segmentation, superpixels were classified using three thresholds for DAB Mean Intensity, according to the pathological precedence of Allred scoring [25], and intensity and shape features were calculated. For vascular-adjacent ROIs, distance to vessel (normalised to the square root of the non-vessel-containing area of the image to account for differences in vessel calibre) and distance to superpixels by threshold class were also calculated. Measurements were exported at the image (number of positive detections per threshold class) and superpixel level (threshold class, intensity, shape, and distance features).

Nuclear and cytoplasmic FUS localisation was quantified using cell segmentation in QuPath. For neuronal quantification, only grey matter ROIs were included, cells were segmented (pixel size = 0.5 µm, background radius = 0, median filter radius = 0, sigma = 4, minimum nuclear area = 150 µm^2^, maximum nuclear area = 5,000 µm^2^, intensity threshold = 0.1, max background intensity = 0, cell expansion = 10 µm) and nuclear, cytoplasmic, and whole cell intensity and shape features were calculated and exported. For glial quantification, both grey and white matter ROIs were included, cells were segmented (pixel size = 0.5 µm, background radius = 0, median filter radius = 0, sigma = 4, minimum nuclear area = 10 µm^2^, maximum nuclear area = 100 µm^2^, intensity threshold = 0.1, max background intensity = 0, cell expansion = 5 µm) and nuclear, cytoplasmic, and whole cell intensity and shape features were calculated and exported. Scripts used in these analyses can be found below.

CD68 NVA

setImageType('BRIGHTFIELD_H_DAB');

setColorDeconvolutionStains('{"Name" : "H-DAB default", "Stain 1" : "Hematoxylin", "Values 1" : "0.65111 0.70119 0.29049 ", "Stain 2" : "DAB", "Values 2" : "0.26917 0.56824 0.77759 ", "Background" : " 255 255 255 "}');

createSelectAllObject(true);

selectAnnotations ();

runPlugin('qupath.imagej.superpixels.DoGSuperpixelsPlugin', '{"downsampleFactor": 1.0, "sigmaPixels": 5.0, "minThreshold": 10.0, "maxThreshold": 230.0, "noiseThreshold": 1.0}');

selectDetections();

runPlugin('qupath.lib.algorithms.IntensityFeaturesPlugin', '{"downsample": 1.0, "region": "ROI", "tileSizePixels": 200.0, "colorOD": false, "colorStain1": false, "colorStain2": true, "colorStain3": false, "colorRed": false, "colorGreen": false, "colorBlue": false, "colorHue": false, "colorSaturation": false, "colorBrightness": false, "doMean": true, "doStdDev": true, "doMinMax": true, "doMedian": true, "doHaralick": false, "haralickDistance": 1, "haralickBins": 32}');

addShapeMeasurements("AREA", "LENGTH", "CIRCULARITY", "SOLIDITY", "MAX_DIAMETER", "MIN_DIAMETER")

setDetectionIntensityClassifications("ROI: 1.00 px per pixel: DAB: Mean", 0.0348, 0.0693, 0.1037)

detectionCentroidDistances(true)

CD68 VA

setImageType('BRIGHTFIELD_H_DAB');

setColorDeconvolutionStains('{"Name" : "H-DAB default", "Stain 1" : "Hematoxylin", "Values 1" : "0.65111 0.70119 0.29049 ", "Stain 2" : "DAB", "Values 2" : "0.26917 0.56824 0.77759 ", "Background" : " 255 255 255 "}');

selectAnnotations ();

def region = getPathClass('Other')

def other = getPathClass('Region*')

getAnnotationObjects().eachWithIndex { annotation , i ->

if (i % 2 == 0)

annotation.setPathClass(region)

else

annotation.setPathClass(other)

}

fireHierarchyUpdate()

makeInverseAnnotation()

runPlugin('qupath.imagej.superpixels.DoGSuperpixelsPlugin', '{"downsampleFactor": 1.0, "sigmaPixels": 5.0, "minThreshold": 10.0, "maxThreshold": 230.0, "noiseThreshold": 1.0}');

selectDetections()

runPlugin('qupath.lib.algorithms.IntensityFeaturesPlugin', '{"downsample": 1.0, "region": "ROI", "tileSizePixels": 200.0, "colorOD": false, "colorStain1": false, "colorStain2": true, "colorStain3": false, "colorRed": false, "colorGreen": false, "colorBlue": false, "colorHue": false, "colorSaturation": false, "colorBrightness": false, "doMean": true, "doStdDev": true, "doMinMax": true, "doMedian": true, "doHaralick": false, "haralickDistance": 1, "haralickBins": 32}');

addShapeMeasurements("AREA", "LENGTH", "CIRCULARITY", "SOLIDITY", "MAX_DIAMETER", "MIN_DIAMETER")

setDetectionIntensityClassifications("ROI: 1.00 px per pixel: DAB: Mean", 0.0348, 0.0693, 0.1037)

selectDetections()

detectionToAnnotationDistances(true)

detectionCentroidDistances(true)

Iba1 NVA

setImageType('BRIGHTFIELD_H_DAB');

setColorDeconvolutionStains('{"Name" : "H-DAB default", "Stain 1" : "Hematoxylin", "Values 1" : "0.65111 0.70119 0.29049 ", "Stain 2" : "DAB", "Values 2" : "0.26917 0.56824 0.77759 ", "Background" : " 255 255 255 "}');

createSelectAllObject(true);

selectAnnotations ();

runPlugin('qupath.imagej.superpixels.DoGSuperpixelsPlugin', '{"downsampleFactor": 1.0, "sigmaPixels": 8.0, "minThreshold": 10.0, "maxThreshold": 230.0, "noiseThreshold": 1.0}');

selectDetections();

runPlugin('qupath.lib.algorithms.IntensityFeaturesPlugin', '{"downsample": 1.0, "region": "ROI", "tileSizePixels": 200.0, "colorOD": false, "colorStain1": false, "colorStain2": true, "colorStain3": false, "colorRed": false, "colorGreen": false, "colorBlue": false, "colorHue": false, "colorSaturation": false, "colorBrightness": false, "doMean": true, "doStdDev": true, "doMinMax": true, "doMedian": true, "doHaralick": false, "haralickDistance": 1, "haralickBins": 32}');

addShapeMeasurements("AREA", "LENGTH", "CIRCULARITY", "SOLIDITY", "MAX_DIAMETER", "MIN_DIAMETER")

setDetectionIntensityClassifications("ROI: 1.00 px per pixel: DAB: Mean", 0.0512, 0.1002, 0.1491)

detectionCentroidDistances(true)

Iba1 VA

setImageType('BRIGHTFIELD_H_DAB');

setColorDeconvolutionStains('{"Name" : "H-DAB default", "Stain 1" : "Hematoxylin", "Values 1" : "0.65111 0.70119 0.29049 ", "Stain 2" : "DAB", "Values 2" : "0.26917 0.56824 0.77759 ", "Background" : " 255 255 255 "}');

selectAnnotations ();

def region = getPathClass('Other')

def other = getPathClass('Region*')

getAnnotationObjects().eachWithIndex { annotation , i ->

if (i % 2 == 0)

annotation.setPathClass(region)

else

annotation.setPathClass(other)

}

fireHierarchyUpdate()

makeInverseAnnotation()

runPlugin('qupath.imagej.superpixels.DoGSuperpixelsPlugin', '{"downsampleFactor": 1.0, "sigmaPixels": 8.0, "minThreshold": 10.0, "maxThreshold": 230.0, "noiseThreshold": 1.0}');

selectDetections()

runPlugin('qupath.lib.algorithms.IntensityFeaturesPlugin', '{"downsample": 1.0, "region": "ROI", "tileSizePixels": 200.0, "colorOD": false, "colorStain1": false, "colorStain2": true, "colorStain3": false, "colorRed": false, "colorGreen": false, "colorBlue": false, "colorHue": false, "colorSaturation": false, "colorBrightness": false, "doMean": true, "doStdDev": true, "doMinMax": true, "doMedian": true, "doHaralick": false, "haralickDistance": 1, "haralickBins": 32}');

addShapeMeasurements("AREA", "LENGTH", "CIRCULARITY", "SOLIDITY", "MAX_DIAMETER", "MIN_DIAMETER")

setDetectionIntensityClassifications("ROI: 1.00 px per pixel: DAB: Mean", 0.0512, 0.1002, 0.1491)

selectDetections()

detectionToAnnotationDistances(true)

detectionCentroidDistances(true)

GFAP NVA

setImageType('BRIGHTFIELD_H_DAB');

setColorDeconvolutionStains('{"Name" : "H-DAB default", "Stain 1" : "Hematoxylin", "Values 1" : "0.65111 0.70119 0.29049 ", "Stain 2" : "DAB", "Values 2" : "0.26917 0.56824 0.77759 ", "Background" : " 255 255 255 "}');

createSelectAllObject(true);

selectAnnotations ();

runPlugin('qupath.imagej.superpixels.DoGSuperpixelsPlugin', '{"downsampleFactor": 1.0, "sigmaPixels": 8.0, "minThreshold": 10.0, "maxThreshold": 230.0, "noiseThreshold": 1.0}');

selectDetections();

runPlugin('qupath.lib.algorithms.IntensityFeaturesPlugin', '{"downsample": 1.0, "region": "ROI", "tileSizePixels": 200.0, "colorOD": false, "colorStain1": false, "colorStain2": true, "colorStain3": false, "colorRed": false, "colorGreen": false, "colorBlue": false, "colorHue": false, "colorSaturation": false, "colorBrightness": false, "doMean": true, "doStdDev": true, "doMinMax": true, "doMedian": true, "doHaralick": false, "haralickDistance": 1, "haralickBins": 32}');

addShapeMeasurements("AREA", "LENGTH", "CIRCULARITY", "SOLIDITY", "MAX_DIAMETER", "MIN_DIAMETER")

setDetectionIntensityClassifications("ROI: 1.00 px per pixel: DAB: Mean", 0.1666, 0.2295, 0.2925)

detectionCentroidDistances(true)

GFAP VA

setImageType('BRIGHTFIELD_H_DAB');

setColorDeconvolutionStains('{"Name" : "H-DAB default", "Stain 1" : "Hematoxylin", "Values 1" : "0.65111 0.70119 0.29049 ", "Stain 2" : "DAB", "Values 2" : "0.26917 0.56824 0.77759 ", "Background" : " 255 255 255 "}');

selectAnnotations ();

def region = getPathClass('Other')

def other = getPathClass('Region*')

getAnnotationObjects().eachWithIndex { annotation , i ->

if (i % 2 == 0)

annotation.setPathClass(region)

else

annotation.setPathClass(other)

}

fireHierarchyUpdate()

makeInverseAnnotation()

runPlugin('qupath.imagej.superpixels.DoGSuperpixelsPlugin', '{"downsampleFactor": 1.0, "sigmaPixels": 8.0, "minThreshold": 10.0, "maxThreshold": 230.0, "noiseThreshold": 1.0}');

selectDetections()

runPlugin('qupath.lib.algorithms.IntensityFeaturesPlugin', '{"downsample": 1.0, "region": "ROI", "tileSizePixels": 200.0, "colorOD": false, "colorStain1": false, "colorStain2": true, "colorStain3": false, "colorRed": false, "colorGreen": false, "colorBlue": false, "colorHue": false, "colorSaturation": false, "colorBrightness": false, "doMean": true, "doStdDev": true, "doMinMax": true, "doMedian": true, "doHaralick": false, "haralickDistance": 1, "haralickBins": 32}');

addShapeMeasurements("AREA", "LENGTH", "CIRCULARITY", "SOLIDITY", "MAX_DIAMETER", "MIN_DIAMETER")

setDetectionIntensityClassifications("ROI: 1.00 px per pixel: DAB: Mean", 0.1666, 0.2295, 0.2925)

selectDetections()

detectionToAnnotationDistances(true)

detectionCentroidDistances(true)

FUS NVA

setImageType('BRIGHTFIELD_H_DAB');

setColorDeconvolutionStains('{"Name" : "H-DAB default", "Stain 1" : "Hematoxylin", "Values 1" : "0.65111 0.70119 0.29049 ", "Stain 2" : "DAB", "Values 2" : "0.26917 0.56824 0.77759 ", "Background" : " 255 255 255 "}');

createSelectAllObject(true);

selectAnnotations ();

runPlugin('qupath.imagej.superpixels.DoGSuperpixelsPlugin', '{"downsampleFactor": 1.0, "sigmaPixels": 5.0, "minThreshold": 10.0, "maxThreshold": 230.0, "noiseThreshold": 1.0}');

selectDetections();

runPlugin('qupath.lib.algorithms.IntensityFeaturesPlugin', '{"downsample": 1.0, "region": "ROI", "tileSizePixels": 200.0, "colorOD": false, "colorStain1": false, "colorStain2": true, "colorStain3": false, "colorRed": false, "colorGreen": false, "colorBlue": false, "colorHue": false, "colorSaturation": false, "colorBrightness": false, "doMean": true, "doStdDev": true, "doMinMax": true, "doMedian": true, "doHaralick": false, "haralickDistance": 1, "haralickBins": 32}');

addShapeMeasurements("AREA", "LENGTH", "CIRCULARITY", "SOLIDITY", "MAX_DIAMETER", "MIN_DIAMETER")

setDetectionIntensityClassifications("ROI: 1.00 px per pixel: DAB: Mean", 0.0900, 0.1100, 0.1300)

detectionCentroidDistances(true)

FUS VA

setImageType('BRIGHTFIELD_H_DAB');

setColorDeconvolutionStains('{"Name" : "H-DAB default", "Stain 1" : "Hematoxylin", "Values 1" : "0.65111 0.70119 0.29049 ", "Stain 2" : "DAB", "Values 2" : "0.26917 0.56824 0.77759 ", "Background" : " 255 255 255 "}');

selectAnnotations ();

def region = getPathClass('Other')

def other = getPathClass('Region*')

getAnnotationObjects().eachWithIndex { annotation , i ->

if (i % 2 == 0)

annotation.setPathClass(region)

else

annotation.setPathClass(other)

}

fireHierarchyUpdate()

makeInverseAnnotation()

runPlugin('qupath.imagej.superpixels.DoGSuperpixelsPlugin', '{"downsampleFactor": 1.0, "sigmaPixels": 5.0, "minThreshold": 10.0, "maxThreshold": 230.0, "noiseThreshold": 1.0}');

selectDetections()

runPlugin('qupath.lib.algorithms.IntensityFeaturesPlugin', '{"downsample": 1.0, "region": "ROI", "tileSizePixels": 200.0, "colorOD": false, "colorStain1": false, "colorStain2": true, "colorStain3": false, "colorRed": false, "colorGreen": false, "colorBlue": false, "colorHue": false, "colorSaturation": false, "colorBrightness": false, "doMean": true, "doStdDev": true, "doMinMax": true, "doMedian": true, "doHaralick": false, "haralickDistance": 1, "haralickBins": 32}');

addShapeMeasurements("AREA", "LENGTH", "CIRCULARITY", "SOLIDITY", "MAX_DIAMETER", "MIN_DIAMETER")

setDetectionIntensityClassifications("ROI: 1.00 px per pixel: DAB: Mean", 0.0900, 0.1100, 0.1300)

selectDetections()

detectionToAnnotationDistances(true)

detectionCentroidDistances(true)

TDP43 NVA

setImageType('BRIGHTFIELD_H_DAB');

setColorDeconvolutionStains('{"Name" : "H-DAB default", "Stain 1" : "Hematoxylin", "Values 1" : "0.65111 0.70119 0.29049 ", "Stain 2" : "DAB", "Values 2" : "0.26917 0.56824 0.77759 ", "Background" : " 255 255 255 "}');

createSelectAllObject(true);

selectAnnotations ();

runPlugin('qupath.imagej.superpixels.DoGSuperpixelsPlugin', '{"downsampleFactor": 1.0, "sigmaPixels": 5.0, "minThreshold": 10.0, "maxThreshold": 230.0, "noiseThreshold": 1.0}');

selectDetections();

runPlugin('qupath.lib.algorithms.IntensityFeaturesPlugin', '{"downsample": 1.0, "region": "ROI", "tileSizePixels": 200.0, "colorOD": false, "colorStain1": false, "colorStain2": true, "colorStain3": false, "colorRed": false, "colorGreen": false, "colorBlue": false, "colorHue": false, "colorSaturation": false, "colorBrightness": false, "doMean": true, "doStdDev": true, "doMinMax": true, "doMedian": true, "doHaralick": false, "haralickDistance": 1, "haralickBins": 32}');

addShapeMeasurements("AREA", "LENGTH", "CIRCULARITY", "SOLIDITY", "MAX_DIAMETER", "MIN_DIAMETER")

setDetectionIntensityClassifications("ROI: 1.00 px per pixel: DAB: Mean", 0.0719, 0.1223, 0.1713)

detectionCentroidDistances(true)

TDP43 VA

setImageType('BRIGHTFIELD_H_DAB');

setColorDeconvolutionStains('{"Name" : "H-DAB default", "Stain 1" : "Hematoxylin", "Values 1" : "0.65111 0.70119 0.29049 ", "Stain 2" : "DAB", "Values 2" : "0.26917 0.56824 0.77759 ", "Background" : " 255 255 255 "}');

selectAnnotations ();

def region = getPathClass('Other')

def other = getPathClass('Region*')

getAnnotationObjects().eachWithIndex { annotation , i ->

if (i % 2 == 0)

annotation.setPathClass(region)

else

annotation.setPathClass(other)

}

fireHierarchyUpdate()

makeInverseAnnotation()

runPlugin('qupath.imagej.superpixels.DoGSuperpixelsPlugin', '{"downsampleFactor": 1.0, "sigmaPixels": 5.0, "minThreshold": 10.0, "maxThreshold": 230.0, "noiseThreshold": 1.0}');

selectDetections()

runPlugin('qupath.lib.algorithms.IntensityFeaturesPlugin', '{"downsample": 1.0, "region": "ROI", "tileSizePixels": 200.0, "colorOD": false, "colorStain1": false, "colorStain2": true, "colorStain3": false, "colorRed": false, "colorGreen": false, "colorBlue": false, "colorHue": false, "colorSaturation": false, "colorBrightness": false, "doMean": true, "doStdDev": true, "doMinMax": true, "doMedian": true, "doHaralick": false, "haralickDistance": 1, "haralickBins": 32}');

addShapeMeasurements("AREA", "LENGTH", "CIRCULARITY", "SOLIDITY", "MAX_DIAMETER", "MIN_DIAMETER")

setDetectionIntensityClassifications("ROI: 1.00 px per pixel: DAB: Mean", 0.0719, 0.1223, 0.1713)

selectDetections()

detectionToAnnotationDistances(true)

detectionCentroidDistances(true)

FUS NVA neurons localisation

setImageType('BRIGHTFIELD_H_DAB');

setColorDeconvolutionStains('{"Name" : "H-DAB default", "Stain 1" : "Hematoxylin", "Values 1" : "0.65111 0.70119 0.29049 ", "Stain 2" : "DAB", "Values 2" : "0.26917 0.56824 0.77759 ", "Background" : " 255 255 255 "}');

setPixelSizeMicrons(0.625,0.625)

createSelectAllObject(true);

selectAnnotations ();

runPlugin('qupath.imagej.detect.cells.WatershedCellDetection', '{"detectionImageBrightfield": "Hematoxylin OD", "requestedPixelSizeMicrons": 0.5, "backgroundRadiusMicrons": 0.0, "medianRadiusMicrons": 0.0, "sigmaMicrons": 4.0, "minAreaMicrons": 150.0, "maxAreaMicrons": 5000.0, "threshold": 0.1, "maxBackground": 2.0, "watershedPostProcess": true, "excludeDAB": false, "cellExpansionMicrons": 10.0, "includeNuclei": true, "smoothBoundaries": true, "makeMeasurements": true}');

FUS VA neurons localisation

setImageType('BRIGHTFIELD_H_DAB');

setColorDeconvolutionStains('{"Name" : "H-DAB default", "Stain 1" : "Hematoxylin", "Values 1" : "0.65111 0.70119 0.29049 ", "Stain 2" : "DAB", "Values 2" : "0.26917 0.56824 0.77759 ", "Background" : " 255 255 255 "}');

setPixelSizeMicrons(0.625,0.625)

selectAnnotations ();

def region = getPathClass('Other')

def other = getPathClass('Region*')

getAnnotationObjects().eachWithIndex { annotation , i ->

if (i % 2 == 0)

annotation.setPathClass(region)

else

annotation.setPathClass(other)

}

fireHierarchyUpdate()

makeInverseAnnotation()

runPlugin('qupath.imagej.detect.cells.WatershedCellDetection', '{"detectionImageBrightfield": "Hematoxylin OD", "requestedPixelSizeMicrons": 0.5, "backgroundRadiusMicrons": 0.0, "medianRadiusMicrons": 0.0, "sigmaMicrons": 4.0, "minAreaMicrons": 150.0, "maxAreaMicrons": 5000.0, "threshold": 0.1, "maxBackground": 2.0, "watershedPostProcess": true, "excludeDAB": false, "cellExpansionMicrons": 10.0, "includeNuclei": true, "smoothBoundaries": true, "makeMeasurements": true}');

FUS VA glial localisation

setImageType('BRIGHTFIELD_H_DAB');

setColorDeconvolutionStains('{"Name" : "H-DAB default", "Stain 1" : "Hematoxylin", "Values 1" : "0.65111 0.70119 0.29049 ", "Stain 2" : "DAB", "Values 2" : "0.26917 0.56824 0.77759 ", "Background" : " 255 255 255 "}');

setPixelSizeMicrons(0.625,0.625)

selectAnnotations ();

def region = getPathClass('Other')

def other = getPathClass('Region*')

getAnnotationObjects().eachWithIndex { annotation , i ->

if (i % 2 == 0)

annotation.setPathClass(region)

else

annotation.setPathClass(other)

}

fireHierarchyUpdate()

makeInverseAnnotation()

runPlugin('qupath.imagej.detect.cells.WatershedCellDetection', '{"detectionImageBrightfield": "Hematoxylin OD", "requestedPixelSizeMicrons": 0.5, "backgroundRadiusMicrons": 0.0, "medianRadiusMicrons": 0.0, "sigmaMicrons": 4.0, "minAreaMicrons": 10.0, "maxAreaMicrons": 100.0, "threshold": 0.1, "maxBackground": 2.0, "watershedPostProcess": true, "excludeDAB": false, "cellExpansionMicrons": 5.0, "includeNuclei": true, "smoothBoundaries": true, "makeMeasurements": true}');

FUS NVA glial localisation

setImageType('BRIGHTFIELD_H_DAB');

setColorDeconvolutionStains('{"Name" : "H-DAB default", "Stain 1" : "Hematoxylin", "Values 1" : "0.65111 0.70119 0.29049 ", "Stain 2" : "DAB", "Values 2" : "0.26917 0.56824 0.77759 ", "Background" : " 255 255 255 "}');

setPixelSizeMicrons(0.625,0.625)

createSelectAllObject(true);

selectAnnotations ();

runPlugin('qupath.imagej.detect.cells.WatershedCellDetection', '{"detectionImageBrightfield": "Hematoxylin OD", "requestedPixelSizeMicrons": 0.5, "backgroundRadiusMicrons": 0.0, "medianRadiusMicrons": 0.0, "sigmaMicrons": 4.0, "minAreaMicrons": 10.0, "maxAreaMicrons": 100.0, "threshold": 0.1, "maxBackground": 2.0, "watershedPostProcess": true, "excludeDAB": false, "cellExpansionMicrons": 5.0, "includeNuclei": true, "smoothBoundaries": true, "makeMeasurements": true}');


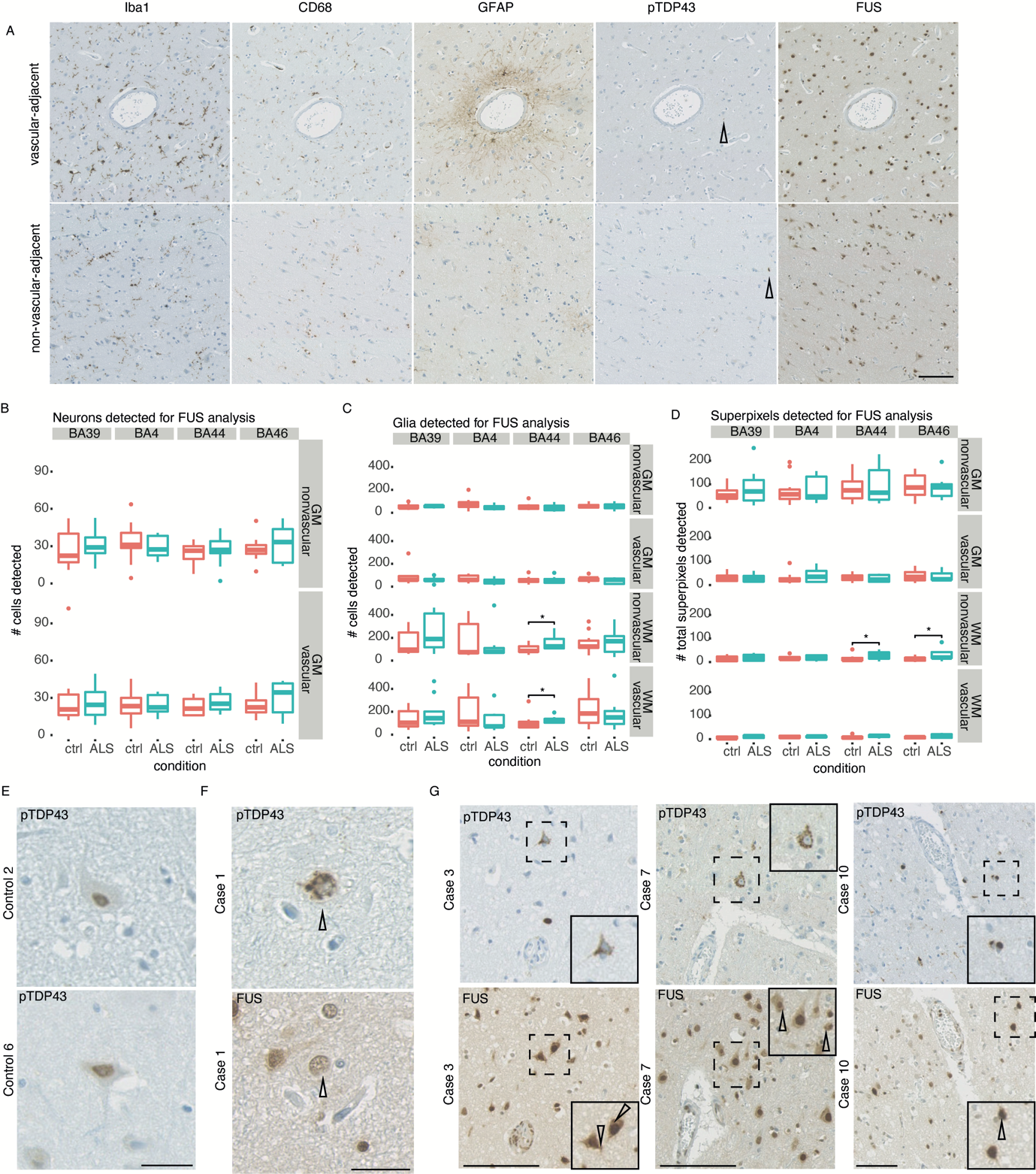


**Figure S1. Supplementary digital pathology analysis information.** (A) Immunohistochemical stains included in digital pathology analysis, showing (top) vascular-adjacent and (bottom) non-vascular adjacent regions. pTDP-43 staining is indicated by arrows. Scale bar, 100 µm. (B) Box and whisker plot showing the number of neurons detected in FUS cell segmentation analysis across all included regions. (C) Box and whisker plot showing the number of glia detected in FUS cell segmentation analysis across all included regions. (D) Box and whisker plot showing the total number of superpixels detected in FUS superpixel segmentation analysis across all included regions. Data in box and whisker plots are averaged across quantified regions not included in the presented stratification, such that each category contains n = number of cases, to avoid pseudoreplication. (E) pTDP-43 immunostaining demonstrating TDP-43 pathology in two control cases. Scale bar, 25 µm. (F) pTDP-43 and FUS immunostaining demonstrating TDP-43 aggregates and nuclear FUS paraspeckles (indicated by arrows) in the same cell across serial sections. Scale bar, 25 µm. (G) pTDP-43 and FUS immunostaining demonstrating TDP-43 and FUS pathology (indicated by arrows) in the same region (indicated by the presence of the same vessel in each image). Scale bars, 100 µm. BA, Brodmann area; GM, grey matter; WM, white matter. Mann–Whitney U test, * p < 0.05, ** p < 0.01, *** p < 0.001, **** p < 0.0001

**Figure S2. Additional glial activation staining.** (a) Box and whisker plot of CD68+ superpixel staining in BA46 between control, two executive-impaired and executive-unimpaired cases. (e) Box and whisker plot of CD68+ superpixel staining in BA44 between control, fluency-unimpaired cases and one fluency-impaired (FTD) case***.*** No statistical tests were conducted for (a) & (b) due to low sample size. (c) Box and whisker plot of GFAP+ superpixel staining in BA39 between control, language-impaired and language-unimpaired cases. (d) Box and whisker plot of GFAP+ superpixel staining in BA4 between control, long and short survivors. Wilcoxon tests with Holm–Šidák multiple comparisons correction were conducted for (c) & (d), with no significant differences found. Data in box and whisker plots are averaged across quantified regions not included in the presented stratification, such that each category contains n = number of cases, in order to avoid pseudoreplication. BA, Brodmann area; GM, grey matter; WM, white matter

# Table S1. Random forest classification of stain panel with sensitivity and specificity scores. Results were considered significant if the number classified correctly was significantly better than chance according to 𝜒^2^ tests.

| by | condition | TRUE | FALSE | EXPECTED | p-value | sensitivity | specificity |
| --- | --- | --- | --- | --- | --- | --- | --- |
| all | control | 527 | 273 | 400 | p < 0.0001 | 0.69 | 0.66 |
|  | disease | 551 | 249 | 400 | p < 0.0001 |  |  |
| CD68 | control | 96 | 64 | 80 | p = 0.0114 | 0.67 | 0.60 |
|  | disease | 107 | 53 | 80 | p < 0.0001 |  |  |
| FUS | control | 111 | 49 | 80 | p < 0.0001 | 0.64 | 0.69 |
|  | disease | 103 | 57 | 80 | p = 0.0003 |  |  |
| GFAP | control | 84 | 76 | 80 | p = 0.527 | 0.79 | 0.53 |
|  | disease | 127 | 33 | 80 | p < 0.0001 |  |  |
| Iba1 | control | 134 | 26 | 80 | p < 0.0001 | 0.78 | 0.84 |
|  | disease | 125 | 35 | 80 | p < 0.0001 |  |  |
| pTDP43 | control | 102 | 58 | 80 | p = 0.0005 | 0.51 | 0.64 |
|  | disease | 81 | 79 | 80 | p = 0.8744 |  |  |
| BA4 | control | 122 | 78 | 100 | p = 0.0019 | 0.79 | 0.61 |
|  | disease | 158 | 42 | 100 | p < 0.0001 |  |  |
| BA39 | control | 136 | 64 | 100 | p < 0.0001 | 0.64 | 0.68 |
|  | disease | 127 | 73 | 100 | p = 0.0001 |  |  |
| BA44 | control | 136 | 64 | 100 | p < 0.0001 | 0.63 | 0.68 |
|  | disease | 126 | 74 | 100 | p = 0.0002 |  |  |
| BA46 | control | 133 | 67 | 100 | p < 0.0001 | 0.66 | 0.67 |
|  | disease | 132 | 68 | 100 | p < 0.0001 |  |  |
| GM | control | 271 | 129 | 200 | p < 0.0001 | 0.68 | 0.68 |
|  | disease | 273 | 127 | 200 | p < 0.0001 |  |  |
| WM | control | 256 | 144 | 200 | p < 0.0001 | 0.68 | 0.64 |
|  | disease | 270 | 130 | 200 | p < 0.0001 |  |  |
| VA | control | 268 | 132 | 200 | p < 0.0001 | 0.65 | 0.67 |
|  | disease | 260 | 140 | 200 | p < 0.0001 |  |  |
| NVA | control | 259 | 141 | 200 | p < 0.0001 | 0.71 | 0.65 |
|  | disease | 283 | 117 | 200 | p < 0.0001 |  |  |

# Table S2. Three-fold cross validation of random forest model. Data were divided equally and randomly into three groups, where each group was considered the test dataset and the other groups considered the training dataset

| Measure | Mean | Standard Deviation |
| --- | --- | --- |
| Accuracy | 0.706 | 0.001 |
| Precision | 0.707 | 0.016 |
| Recall | 0.704 | 0.013 |

# Table S3. Random forest leave-one-out feature analysis. List of features that when left out of the random forest model worsened predictivity of disease status.

| Condition | TRUE | FALSE | Iteration | Feature | Improvement (%) |
| --- | --- | --- | --- | --- | --- |
| disease | 542 | 258 | 36 | Average minimum diameter (px) of class 2+ superpixels | -0.1841621 |
| disease | 542 | 258 | 45 | Average maximum diameter (px) of class 3+ superpixels | -0.1841621 |
| disease | 542 | 258 | 59 | Average distance (px) of negative superpixels to class 2+ superpixels | -0.1841621 |
| disease | 542 | 258 | 88 | Average glial nuclear haematoxylin intensity (range) | -0.1841621 |
| disease | 542 | 258 | 89 | Average glial nuclear DAB intensity (mean) | -0.1841621 |
| disease | 542 | 258 | 90 | Average glial nuclear DAB intensity (sum) | -0.1841621 |
| disease | 542 | 258 | 92 | Average glial nuclear DAB intensity (max) | -0.1841621 |
| disease | 542 | 258 | 95 | Average glial cell area | -0.1841621 |
| disease | 542 | 258 | 147 | Average neuronal cell DAB intensity (mean) | -0.1841621 |
| disease | 541 | 259 | 37 | Average distance (px) of class 2+ superpixels to negative superpixels | -0.3683241 |
| disease | 541 | 259 | 43 | Average circularity of class 3+ superpixels | -0.3683241 |
| disease | 541 | 259 | 85 | Average glial nuclear haematoxylin intensity (standard deviation) | -0.3683241 |
| disease | 541 | 259 | 116 | Average glial cytoplasmic DAB intensity (minimum) | -0.3683241 |
| disease | 541 | 259 | 117 | Average glial nucleus/cell area ratio | -0.3683241 |
| disease | 541 | 259 | 140 | Average neuronal cell maximum caliper | -0.3683241 |
| disease | 541 | 259 | 142 | Average neuronal cell eccentricity | -0.3683241 |
| disease | 541 | 259 | 153 | Average neuronal cytoplasmic haematoxylin intensity (max) | -0.3683241 |
| disease | 541 | 259 | 160 | Average neuronal nuclear/cytoplasmic DAB intensity ratio | -0.3683241 |
| disease | 540 | 260 | 4 | Vessel Allred score (vascular-adjacent images) | -0.5524862 |
| disease | 540 | 260 | 40 | Average distance (px) of class 2+ superpixels to class 3+ superpixels | -0.5524862 |
| disease | 540 | 260 | 63 | Average normalised distance (px) of class 3+ superpixels to vessel (vascular-adjacent images) | -0.5524862 |
| disease | 540 | 260 | 71 | Area of vessel retraction (%) (vascular-adjacent images) | -0.5524862 |
| disease | 540 | 260 | 91 | Average glial nuclear DAB intensity (standard deviation) | -0.5524862 |
| disease | 539 | 261 | 18 | Percent of superpixels that are class 2+ | -0.7366483 |
| disease | 539 | 261 | 23 | Average circularity of class 1+ superpixels | -0.7366483 |
| disease | 539 | 261 | 54 | Average solidity of negative superpixels | -0.7366483 |
| disease | 539 | 261 | 61 | Average normalised distance (px) of class 1+ superpixels to vessel (vascular-adjacent images) | -0.7366483 |
| disease | 539 | 261 | 110 | Average glial cytoplasmic haematoxylin intensity (standard deviation) | -0.7366483 |
| disease | 539 | 261 | 113 | Average glial cytoplasmic DAB intensity (mean) | -0.7366483 |
| disease | 539 | 261 | 115 | Average glial cytoplasmic DAB intensity (max) | -0.7366483 |
| disease | 539 | 261 | 141 | Average neuronal cell minimum caliper | -0.7366483 |
| disease | 539 | 261 | 152 | Average neuronal cytoplasmic haematoxylin intensity (standard deviation) | -0.7366483 |
| disease | 538 | 262 | 12 | Image Allred proportion | -0.9208103 |
| disease | 538 | 262 | 30 | Average distance (px) of class 1+ superpixels to class 3+ superpixels | -0.9208103 |
| disease | 538 | 262 | 42 | Average length (px) of class 3+ superpixels | -0.9208103 |
| disease | 538 | 262 | 84 | Average glial nuclear haematoxylin intensity (sum) | -0.9208103 |
| disease | 538 | 262 | 93 | Average glial nuclear DAB intensity (min) | -0.9208103 |
| disease | 538 | 262 | 98 | Average glial cell maximum caliper | -0.9208103 |
| disease | 538 | 262 | 101 | Average glial cell haematoxylin intensity (mean) | -0.9208103 |
| disease | 538 | 262 | 107 | Average glial cell DAB intensity (max) | -0.9208103 |
| disease | 538 | 262 | 108 | Average glial cell DAB intensity (min) | -0.9208103 |
| disease | 538 | 262 | 112 | Average glial cytoplasmic haematoxylin intensity (min) | -0.9208103 |
| disease | 538 | 262 | 132 | Average neuronal nuclear DAB intensity (sum) | -0.9208103 |
| disease | 538 | 262 | 134 | Average neuronal nuclear DAB intensity (max) | -0.9208103 |
| disease | 537 | 263 | 7 | Percent of superpixels that are class 1+ in vessel (vascular-adjacent images) | -1.1049724 |
| disease | 537 | 263 | 24 | Average solidity of class 1+ superpixels | -1.1049724 |
| disease | 537 | 263 | 31 | Average area of class 2+ superpixels | -1.1049724 |
| disease | 537 | 263 | 35 | Average maximum diameter (px) of class 2+ superpixels | -1.1049724 |
| disease | 537 | 263 | 49 | Average distance (px) of class 2+ superpixels to class 3+ superpixels | -1.1049724 |
| disease | 537 | 263 | 50 | Average distance (px) of class 3+ superpixels to class 3+ superpixels | -1.1049724 |
| disease | 537 | 263 | 53 | Average circularity of negative detections | -1.1049724 |
| disease | 537 | 263 | 58 | Average distance (px) of negative superpixels to class 1+ superpixels | -1.1049724 |
| disease | 537 | 263 | 66 | Vessel H-score (vascular-adjacent images) | -1.1049724 |
| disease | 537 | 263 | 83 | Average glial nuclear haematoxylin intensity (mean) | -1.1049724 |
| disease | 537 | 263 | 86 | Average glial nuclear haematoxylin intensity (max) | -1.1049724 |
| disease | 537 | 263 | 106 | Average glial cell DAB intensity (standard deviation) | -1.1049724 |
| disease | 537 | 263 | 127 | Average neuronal nuclear haematoxylin intensity (standard deviation) | -1.1049724 |
| disease | 537 | 263 | 148 | Average neuronal cell DAB intensity (standard deviation) | -1.1049724 |
| disease | 537 | 263 | 157 | Average neuronal cytoplasmic DAB intensity (max) | -1.1049724 |
| disease | 536 | 264 | 1 | Vessel H-score (vascular-adjacent images) | -1.2891344 |
| disease | 536 | 264 | 8 | Percent of superpixels that are class 2+ in vessel (vascular-adjacent images) | -1.2891344 |
| disease | 536 | 264 | 11 | Image H-score | -1.2891344 |
| disease | 536 | 264 | 19 | Percent of superpixels that are class 3+ | -1.2891344 |
| disease | 536 | 264 | 25 | Average maximum diameter of class 1+ superpixels | -1.2891344 |
| disease | 536 | 264 | 47 | Average distance of class 3+ superpixels to negative superpixels | -1.2891344 |
| disease | 536 | 264 | 48 | Average distance (px) of class 3+ superpixels to class 1+ superpixels | -1.2891344 |
| disease | 536 | 264 | 68 | Vessel Allred intensity (vascular-adjacent images) | -1.2891344 |
| disease | 536 | 264 | 70 | Vessel area (vascular-adjacent images) | -1.2891344 |
| disease | 536 | 264 | 72 | Percent vessel retraction (vascular-adjacent images) | -1.2891344 |
| disease | 536 | 264 | 100 | Average glial cell eccentricity | -1.2891344 |
| disease | 536 | 264 | 120 | Average neuronal nuclear perimeter | -1.2891344 |
| disease | 536 | 264 | 121 | Average neuronal nuclear circularity | -1.2891344 |
| disease | 536 | 264 | 129 | Average neuronal nuclear haematoxylin intensity (min) | -1.2891344 |
| disease | 536 | 264 | 154 | Average neuronal cytoplasmic haematoxylin intensity (min) | -1.2891344 |
| disease | 535 | 265 | 0 | Percent of superpixels that are positive in vessel (vascular-adjacent images) | -1.4732965 |
| disease | 535 | 265 | 3 | Vessel Allred intensity (vascular-adjacent images) | -1.4732965 |
| disease | 535 | 265 | 22 | Average length (px) of class 1+ superpixels | -1.4732965 |
| disease | 535 | 265 | 41 | Average area (px^2) of class 3+ superpixels | -1.4732965 |
| disease | 535 | 265 | 51 | Average area (px^2) of negative superpixels | -1.4732965 |
| disease | 535 | 265 | 55 | Average maximum diameter (px) of negative superpixels | -1.4732965 |
| disease | 535 | 265 | 73 | Percent of superpixels that are class 1+ in vessel (vascular-adjacent images) | -1.4732965 |
| disease | 535 | 265 | 77 | Average glial nuclear area | -1.4732965 |
| disease | 535 | 265 | 81 | Average glial nuclear minimum caliper | -1.4732965 |
| disease | 535 | 265 | 82 | Average glial nuclear eccentricity | -1.4732965 |
| disease | 535 | 265 | 97 | Average glial cell circularity | -1.4732965 |
| disease | 535 | 265 | 126 | Average neuronal nuclear haematoxylin intensity (sum) | -1.4732965 |
| disease | 535 | 265 | 128 | Average neuronal nuclear haematoxylin intensity (max) | -1.4732965 |
| disease | 535 | 265 | 130 | Average neuronal nuclear haematoxylin intensity (range) | -1.4732965 |
| disease | 535 | 265 | 131 | Average neuronal nuclear DAB intensity (mean) | -1.4732965 |
| disease | 534 | 266 | 6 | Perimeter (px) of vessel (vascular-adjacent images) | -1.6574586 |
| disease | 534 | 266 | 9 | Percent of superpixels that are class 3+ in vessel (vascular-adjacent images) | -1.6574586 |
| disease | 534 | 266 | 15 | Image area excluding vessel (px^2) (vascular-adjacent images) | -1.6574586 |
| disease | 534 | 266 | 16 | Vessel perimeter (px) (vascular-adjacent images) | -1.6574586 |
| disease | 534 | 266 | 26 | Average minimum diameter (px) of class 1+ superpixels | -1.6574586 |
| disease | 534 | 266 | 57 | Average distance (px) of negative superpixels to negative superpixels | -1.6574586 |
| disease | 534 | 266 | 75 | Percent of superpixels that are class 3+ in vessel (vascular-adjacent images) | -1.6574586 |
| disease | 534 | 266 | 79 | Average glial nuclear circularity | -1.6574586 |
| disease | 534 | 266 | 102 | Average glial cell haematoxylin intensity (standard deviation) | -1.6574586 |
| disease | 534 | 266 | 104 | Average glial cell haematoxylin intensity (min) | -1.6574586 |
| disease | 534 | 266 | 114 | Average glial cytoplasmic DAB intensity (standard deviation) | -1.6574586 |
| disease | 534 | 266 | 135 | Average neuronal nuclear DAB intensity (min) | -1.6574586 |
| disease | 534 | 266 | 137 | Average neuronal cell area | -1.6574586 |
| disease | 534 | 266 | 149 | Average neuronal cell DAB intensity (max) | -1.6574586 |
| disease | 534 | 266 | 158 | Average neuronal cytoplasmic DAB intensity (min) | -1.6574586 |
| disease | 533 | 267 | 2 | Vessel Allred proportion | -1.8416206 |
| disease | 533 | 267 | 29 | Average distance (px) of class 1+ superpixels to class 2+ superpixels | -1.8416206 |
| disease | 533 | 267 | 38 | Average distance (px) of class 2+ superpixels to class 1+ superpixels | -1.8416206 |
| disease | 533 | 267 | 52 | Average length (px) of negative superpixels | -1.8416206 |
| disease | 533 | 267 | 151 | Average neuronal cytoplasmic haematoxylin intensity (mean) | -1.8416206 |
| disease | 533 | 267 | 156 | Average neuronal cytoplasmic DAB intensity (standard deviation) | -1.8416206 |
| disease | 532 | 268 | 5 | Area (px^2) of vessel (vascular-adjacent images) | -2.0257827 |
| disease | 532 | 268 | 27 | Average distance (px) of class 1+ superpixels to negative superpixels | -2.0257827 |
| disease | 532 | 268 | 28 | Average distance (px) of class 1+ superpixels to class 1+ superpixels | -2.0257827 |
| disease | 532 | 268 | 56 | Average minimum diameter (px) of negative superpixels | -2.0257827 |
| disease | 532 | 268 | 74 | Percent of superpixels that are class 2+ in vessel (vascular-adjacent images) | -2.0257827 |
| disease | 532 | 268 | 78 | Average glial nuclear perimeter | -2.0257827 |
| disease | 532 | 268 | 80 | Average glial nuclear maximum caliper | -2.0257827 |
| disease | 532 | 268 | 99 | Average glial cell minimum caliper | -2.0257827 |
| disease | 532 | 268 | 122 | Average neuronal nuclear maximum caliper | -2.0257827 |
| disease | 531 | 269 | 10 | Percent superpixels that are positive | -2.2099448 |
| disease | 531 | 269 | 13 | Image Allred intensity | -2.2099448 |
| disease | 531 | 269 | 65 | Percent of superpixels that are positive in vessel (vascular-adjacent images) | -2.2099448 |
| disease | 531 | 269 | 109 | Average glial cytoplasmic haematoxylin intensity (range) | -2.2099448 |
| disease | 531 | 269 | 119 | Average neuronal nuclear area | -2.2099448 |
| disease | 530 | 270 | 14 | Image Allred score | -2.3941068 |
| disease | 530 | 270 | 33 | Average circularity of class 2+ superpixels | -2.3941068 |
| disease | 530 | 270 | 105 | Average glial cell DAB intensity (mean) | -2.3941068 |
| disease | 530 | 270 | 118 | Average glial nuclear/cytoplasmic DAB intensity ratio | -2.3941068 |
| disease | 529 | 271 | 123 | Average neuronal nuclear minimum caliper | -2.5782689 |
| disease | 529 | 271 | 136 | Average neuronal nuclear DAB intensity (range) | -2.5782689 |
| disease | 528 | 272 | 34 | Average solidity of class 2+ superpixels | -2.7624309 |
| disease | 528 | 272 | 69 | Vessel Allred score | -2.7624309 |
| disease | 528 | 272 | 103 | Average glial cell haematoxylin intensity (max) | -2.7624309 |
| disease | 527 | 273 | 17 | Percent of superpixels that are class 1+ | -2.946593 |
| disease | 527 | 273 | 67 | Vessel Allred proportion | -2.946593 |
| disease | 525 | 275 | 64 | Average normalised distance (px) of negative superpixels to vessel (vascular-adjacent images) | -3.3149171 |
| disease | 525 | 275 | 124 | Average neuronal nuclear eccentricity | -3.3149171 |
| disease | 524 | 276 | 125 | Average neuronal haematoxylin intensity (mean) | -3.4990792 |

List of features that reduced predictiveness when removed from the random forest model as part of leave-one-out analysis. Haematoxylin and DAB intensity measures are in units of optical density (OD). Features are colour-coded into DAB superpixel staining (Iba1, CD68, GFAP, pTDP-43, FUS) (yellow), DAB cell staining (FUS) (purple), vessel staining & morphology (green), superpixel morphology (orange), haematoxylin cell staining & morphology (blue), and superpixel spatial distribution (pink) groups. Features may appear twice in different iterations if they were part of multiple datasets (image-level, superpixel-level, vessel retraction-level). Of the 138 listed features there were 9 superpixel staining, 26 cell (FUS) staining, 21 vessel-related, 19 superpixel morphology, 44 cell morphology, and 17 superpixel spatial distribution features. Of the top 50 most predictive features, there were 5 (10%) superpixel staining, 9 (18%) cell (FUS) staining, 11 (22%) vessel-related, 4 (8%) superpixel morphology, 15 (30%) cell morphology, and 6 (12%) superpixel spatial distribution features.

# Table S4. Random forest features included in the model. List of feature types included in the random forest model. Features are colour-coded into DAB superpixel staining (Iba1, CD68, GFAP, pTDP-43, FUS) (yellow), DAB cell staining (FUS) (purple), vessel staining & morphology (green), superpixel morphology (orange), haematoxylin cell staining & morphology (blue), and superpixel spatial distribution (pink) groups. Features may appear twice in different iterations if they were part of multiple datasets (image-level, superpixel-level, vessel retraction-level).

| Feature |
| --- |
| Area (px^2) of vessel (vascular-adjacent images) |
| Area of vessel retraction (%) (vascular-adjacent images) |
| Average area (px^2) of class 1+ superpixels |
| Average area (px^2) of class 3+ superpixels |
| Average area (px^2) of negative superpixels |
| Average area of class 2+ superpixels |
| Average circularity of class 1+ superpixels |
| Average circularity of class 2+ superpixels |
| Average circularity of class 3+ superpixels |
| Average circularity of negative detections |
| Average distance (px) of class 1+ superpixels to class 1+ superpixels |
| Average distance (px) of class 1+ superpixels to class 2+ superpixels |
| Average distance (px) of class 1+ superpixels to class 3+ superpixels |
| Average distance (px) of class 1+ superpixels to negative superpixels |
| Average distance (px) of class 2+ superpixels to class 1+ superpixels |
| Average distance (px) of class 2+ superpixels to class 2+ superpixels |
| Average distance (px) of class 2+ superpixels to class 3+ superpixels |
| Average distance (px) of class 2+ superpixels to class 3+ superpixels |
| Average distance (px) of class 2+ superpixels to negative superpixels |
| Average distance (px) of class 3+ superpixels to class 1+ superpixels |
| Average distance (px) of class 3+ superpixels to class 3+ superpixels |
| Average distance (px) of negative superpixels to class 1+ superpixels |
| Average distance (px) of negative superpixels to class 2+ superpixels |
| Average distance (px) of negative superpixels to class 3+ superpixels |
| Average distance (px) of negative superpixels to negative superpixels |
| Average distance of class 3+ superpixels to negative superpixels |
| Average glial cell area |
| Average glial cell circularity |
| Average glial cell DAB intensity (max) |
| Average glial cell DAB intensity (mean) |
| Average glial cell DAB intensity (min) |
| Average glial cell DAB intensity (standard deviation) |
| Average glial cell eccentricity |
| Average glial cell haematoxylin intensity (max) |
| Average glial cell haematoxylin intensity (mean) |
| Average glial cell haematoxylin intensity (min) |
| Average glial cell haematoxylin intensity (standard deviation) |
| Average glial cell maximum caliper |
| Average glial cell minimum caliper |
| Average glial cell perimeter |
| Average glial cytoplasmic DAB intensity (max) |
| Average glial cytoplasmic DAB intensity (mean) |
| Average glial cytoplasmic DAB intensity (minimum) |
| Average glial cytoplasmic DAB intensity (standard deviation) |
| Average glial cytoplasmic haematoxylin intensity (max) |
| Average glial cytoplasmic haematoxylin intensity (min) |
| Average glial cytoplasmic haematoxylin intensity (range) |
| Average glial cytoplasmic haematoxylin intensity (standard deviation) |
| Average glial nuclear area |
| Average glial nuclear circularity |
| Average glial nuclear DAB intensity (max) |
| Average glial nuclear DAB intensity (mean) |
| Average glial nuclear DAB intensity (min) |
| Average glial nuclear DAB intensity (range) |
| Average glial nuclear DAB intensity (standard deviation) |
| Average glial nuclear DAB intensity (sum) |
| Average glial nuclear eccentricity |
| Average glial nuclear haematoxylin intensity (max) |
| Average glial nuclear haematoxylin intensity (mean) |
| Average glial nuclear haematoxylin intensity (min) |
| Average glial nuclear haematoxylin intensity (range) |
| Average glial nuclear haematoxylin intensity (standard deviation) |
| Average glial nuclear haematoxylin intensity (sum) |
| Average glial nuclear maximum caliper |
| Average glial nuclear minimum caliper |
| Average glial nuclear perimeter |
| Average glial nuclear/cytoplasmic DAB intensity ratio |
| Average glial nucleus/cell area ratio |
| Average length (px) of class 1+ superpixels |
| Average length (px) of class 2+ superpixels |
| Average length (px) of class 3+ superpixels |
| Average length (px) of negative superpixels |
| Average maximum diameter (px) of class 2+ superpixels |
| Average maximum diameter (px) of class 3+ superpixels |
| Average maximum diameter (px) of negative superpixels |
| Average maximum diameter (px) of class 1+ superpixels |
| Average minimum diameter (px) of class 1+ superpixels |
| Average minimum diameter (px) of class 2+ superpixels |
| Average minimum diameter (px) of class 3+ superpixels |
| Average minimum diameter (px) of negative superpixels |
| Average neuronal cell area |
| Average neuronal cell circularity |
| Average neuronal cell DAB intensity (max) |
| Average neuronal cell DAB intensity (mean) |
| Average neuronal cell DAB intensity (min) |
| Average neuronal cell DAB intensity (standard deviation) |
| Average neuronal cell eccentricity |
| Average neuronal cell haematoxylin intensity (max) |
| Average neuronal cell haematoxylin intensity (mean) |
| Average neuronal cell haematoxylin intensity (min) |
| Average neuronal cell haematoxylin intensity (standard deviation) |
| Average neuronal cell maximum caliper |
| Average neuronal cell minimum caliper |
| Average neuronal cell perimeter |
| Average neuronal cytoplasmic DAB intensity (max) |
| Average neuronal cytoplasmic DAB intensity (mean) |
| Average neuronal cytoplasmic DAB intensity (min) |
| Average neuronal cytoplasmic DAB intensity (standard deviation) |
| Average neuronal cytoplasmic haematoxylin intensity (max) |
| Average neuronal cytoplasmic haematoxylin intensity (mean) |
| Average neuronal cytoplasmic haematoxylin intensity (min) |
| Average neuronal cytoplasmic haematoxylin intensity (standard deviation) |
| Average neuronal haematoxylin intensity (mean) |
| Average neuronal nuclear area |
| Average neuronal nuclear cell area ratio |
| Average neuronal nuclear circularity |
| Average neuronal nuclear DAB intensity (max) |
| Average neuronal nuclear DAB intensity (mean) |
| Average neuronal nuclear DAB intensity (min) |
| Average neuronal nuclear DAB intensity (range) |
| Average neuronal nuclear DAB intensity (standard deviation) |
| Average neuronal nuclear DAB intensity (sum) |
| Average neuronal nuclear eccentricity |
| Average neuronal nuclear haematoxylin intensity (max) |
| Average neuronal nuclear haematoxylin intensity (min) |
| Average neuronal nuclear haematoxylin intensity (range) |
| Average neuronal nuclear haematoxylin intensity (standard deviation) |
| Average neuronal nuclear haematoxylin intensity (sum) |
| Average neuronal nuclear maximum caliper |
| Average neuronal nuclear minimum caliper |
| Average neuronal nuclear perimeter |
| Average neuronal nuclear/cytoplasmic DAB intensity ratio |
| Average normalised distance (px) of class 1+ superpixels to vessel (vascular-adjacent images) |
| Average normalised distance (px) of class 2+ superpixels to vessel (vascular-adjacent images) |
| Average normalised distance (px) of class 3+ superpixels to vessel (vascular-adjacent images) |
| Average normalised distance (px) of negative superpixels to vessel (vascular-adjacent images) |
| Average solidity of class 1+ superpixels |
| Average solidity of class 2+ superpixels |
| Average solidity of class 3+ superpixels |
| Average solidity of negative superpixels |
| Image Allred intensity |
| Image Allred proportion |
| Image Allred score |
| Image area excluding vessel (px^2) (vascular-adjacent images) |
| Image H-score |
| Percent of superpixels that are class 1+ |
| Percent of superpixels that are class 1+ in vessel (vascular-adjacent images) |
| Percent of superpixels that are class 1+ in vessel (vascular-adjacent images) |
| Percent of superpixels that are class 2+ |
| Percent of superpixels that are class 2+ in vessel (vascular-adjacent images) |
| Percent of superpixels that are class 2+ in vessel (vascular-adjacent images) |
| Percent of superpixels that are class 3+ |
| Percent of superpixels that are class 3+ in vessel (vascular-adjacent images) |
| Percent of superpixels that are class 3+ in vessel (vascular-adjacent images) |
| Percent of superpixels that are positive in vessel (vascular-adjacent images) |
| Percent of superpixels that are positive in vessel (vascular-adjacent images) |
| Percent superpixels that are positive |
| Percent vessel retraction (vascular-adjacent images) |
| Perimeter (px) of vessel (vascular-adjacent images) |
| Vessel Allred intensity (vascular-adjacent images) |
| Vessel Allred intensity (vascular-adjacent images) |
| Vessel Allred proportion |
| Vessel Allred proportion |
| Vessel Allred score |
| Vessel Allred score (vascular-adjacent images) |
| Vessel area (vascular-adjacent images) |
| Vessel H-score (vascular-adjacent images) |
| Vessel H-score (vascular-adjacent images) |
| Vessel perimeter (px) (vascular-adjacent images) |
